# Transition from acute kidney injury to chronic kidney disease: molecular mechanisms and therapeutic interventions

**DOI:** 10.1186/s43556-026-00462-z

**Published:** 2026-05-09

**Authors:** Xinyue Huang, Mengqiong Wang, Binfeng Yu, Pingping Ren, Xueyan Bian, Ping Wang, Xi Yao, Weiqiang Lin

**Affiliations:** 1 Department of Urology, Center for Oncology Medicine, The Fourth Affiliated Hospital of School of Medicine, and International School of Medicine, International Institutes of Medicine, Zhejiang University, Yiwu, Zhejiang 322000 China; 2 Zhejiang Key Laboratory of Precision Diagnosis and Treatment for Lung Cancer, Yiwu, Zhejiang 322000 China; 3 Department of Liver and Infectious Disease, Sir Run Run Shaw Hospital, Zhejiang University School of Medicine, Hangzhou, Zhejiang 310016 China; 4 Kidney Disease Center, The First Affiliated Hospital, Zhejiang University School of Medicine, Hangzhou, Zhejiang 310003 China; 5 Jiangxi Hospital, The First Affiliated Hospital, Zhejiang University School of Medicine, Nanchang, Jiangxi 330006 China; 6 Department of Nephrology, The First Affiliated Hospital of Ningbo University, Ningbo, Zhejiang 315010 China; 7 Department of Urology, The First Affiliated Hospital, Zhejiang University School of Medicine, Hangzhou, Zhejiang 310003 China

**Keywords:** AKI-CKD transition, Renal fibrosis, Biomarkers, Therapeutic intervention

## Abstract

Acute kidney injury (AKI) is now more frequently recognized as a crucial driver of chronic kidney disease (CKD) rather than merely a curable clinical event. The progression from AKI to CKD (AKI-CKD) mainly results from inadequate adaptive repair mechanisms, which cause ongoing structural damage and a gradual deterioration in kidney function. Here, we systematically dissect the pathological mechanisms underlying this process, with particular emphasis on persistent cell cycle arrest, cellular senescence, chronic infiltration of immune cells, and capillary rarefaction. Leveraging recent advancements in single-cell and spatial omics, we highlight how distinct tubular epithelial cell (TEC) states and aberrant cell–cell interactions orchestrate a profibrotic niche marked by prolonged fibroblast activation and excessive accumulation of extracellular matrix (ECM). Central to these processes is the improper activation of crucial signaling pathways, such as transforming growth factor β (TGF-β)/suppressor of mothers against decapentaplegic homolog (Smad), Wnt/β-catenin, and Hedgehog signaling, accompanied by profound metabolic reprogramming and epigenetic remodeling. We also summarize emerging biomarkers, and strategies enabled by imaging and omics technologies for early diagnosis and risk stratification. Finally, we discuss therapeutic interventions targeting maladaptive signaling networks, inflammatory circuits, and fibrotic pathways to promote adaptive regeneration and prevent CKD progression. We aim to provide insights into improving long-term renal prognosis with a comprehensive examination of the pathological mechanism, diagnostic approaches and targeted interventions associated with the AKI-CKD transition.

## Introduction

Acute kidney injury (AKI) refers to an abrupt decline in renal filtration capacity occurring within 7 days or less [1]. Its frequent occurrence and complicated clinical outcomes make AKI a significant challenge in the field of clinical nephrology [2–4]. While the kidney possesses a natural capacity to recover, a proportion of survivors face a considerably increased risk of progression to chronic kidney disease (CKD) and end-stage renal disease (ESRD), thereby imposing substantial burdens on both society and patients [5]. The transition from AKI to CKD (AKI-CKD) is influenced by factors such as the etiology, intensity, and duration of the initial damage, as well as comorbidities such as diabetes and hypertension [6–10]. Concurrently, individuals with CKD are more susceptible to AKI [11]. An acute insult often triggers a prolonged and irreversible process of chronic renal deterioration. This transition is not merely a clinical outcome [5], but rather a pathophysiological process driven by coordinated cellular and molecular events. The hallmark of this progression is the predominance of maladaptive repair over adaptive repair mechanisms [12]. In response to mild injury, adaptive repair promotes renal recovery, and is manifested by tubular epithelial cell (TEC) remodeling, the timely resolution of inflammation, and the normalization of injury biomarkers [13]. In contrast, the consequences of maladaptive repair cause not only cell cycle arrest and senescence in TECs but also the disruption of the renal interstitial microenvironment. This process includes persistent inflammatory cell infiltration and sustained activation of myofibroblasts, together with chronic hypoxia resulting from capillary rarefaction. Collectively, these changes establish a profibrotic microenvironment that drives CKD progression [12, 14]. Therefore, identification of key molecular nodes in AKI-CKD transition holds significant clinical importance for preventing fibrosis progression and improving renal outcomes.

The diagnosis of AKI and CKD primarily relies on traditional indicators, including serum creatinine (SCr), urine output and estimated glomerular filtration rate (eGFR). However, these indicators lag behind renal parenchymal and pathophysiological changes, and therefore fail to detect injury early [15]. This limitation is particularly evident in subclinical AKI, where pathological alterations occur despite stable SCr and eGFR levels [16]. Several novel biomarkers, including neutrophil gelatinase-associated lipocalin (NGAL), kidney injury molecule-1 (KIM-1), and soluble urokinase-type plasminogen activator receptor (suPAR) have demonstrated superior sensitivity and specificity for the clinical diagnosis of AKI [17]. Furthermore, emerging research suggests that these biomarkers possess predictive value for the risk of CKD progression following AKI [18]. Early and accurate detection is essential for identifying persistent injury and long-term risk, thereby enabling timely intervention to prevent the AKI-CKD transition. Currently, AKI treatment remains largely supportive [2, 19]. While established CKD therapies such as renin angiotensin aldosterone system (RAAS) blockers, sodium-glucose cotransporter-2 inhibitors (SGLT2i), and nonsteroidal mineralocorticoid receptor antagonists (ns-MRAs) mitigate chronic progression [20], specific interventions targeting the AKI-CKD transition phase remain elusive. Although novel therapies show promise in preclinical models, translating these findings into clinical efficacy remains a significant hurdle requiring rigorous validation.

Recent advances in single-cell and omics technologies have remarkably revolutionized our views of cellular heterogeneity, cell–cell interactions, and microenvironmental remodeling during the AKI-CKD transition. By integrating these technological advances, this review establishes a comprehensive framework for understanding how acute injury evolves into chronic disease. We first systematically elucidate the pathophysiological basis of this process, encompassing cellular senescence, inflammatory niche construction, fibroblast activation, and microvascular rarefaction. We then discuss the core regulatory pathways and highlight recent breakthroughs in metabolic remodeling and epigenetic memory that sustain profibrotic phenotypes. Finally, we summarize emerging biomarkers for risk stratification and explore innovative therapeutic strategies to promote adaptive repair and attenuate fibrosis.

## Pathophysiological basis of the AKI-CKD transition

**Fig. 1:**
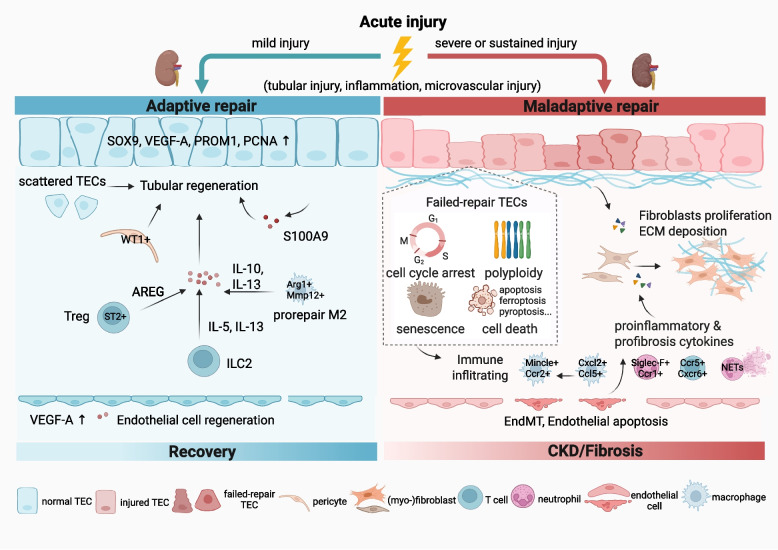
Divergent repair trajectories following AKI and cell events driving the AKI-CKD transition. Mild injury typically triggers an adaptive repair program. TECs upregulate progenitor markers such as SOX9, together with ST2 + Treg and ILC2 cells, secrete prorepair factors to support tubular and microvascular regeneration, restoring renal function. In contrast, severe or sustained injury causes maladaptive repair. TECs exhibit G2/M arrest, polyploidization, senescence, and cell death, releasing proinflammatory and profibrotic mediators. These promote the recruitment of immune cells, induces EndMT, activates fibroblasts, and drives ECM deposition, leading to renal fibrosis and progression to CKD. AKI acute kidney injury; AREG amphiregulin; CKD chronic kidney disease; ECM extracellular matrix; EndMT endothelial-to-mesenchymal transition; IL5/10/13 interleukin 5/10/13; ILC2 type 2 innate lymphoid cell; NETs neutrophil extracellular traps; PROM1 prominin 1; ST2 suppression of tumorigenicity 2; TECs tubular epithelial cells; VEGF-A vascular endothelial growth factor A; Ccr1/2/5 C–C chemokine receptor type 1/2/5; Cxcr6 C-X-C chemokine receptor type 6; Cxcl2 C-X-C motif chemokine ligand 2; Mincle macrophage inducible C-type lectin; Siglec-F sialic acid-binding immunoglobulin-type lectin F; Mmp12 matrix metalloproteinase-12; TEC Tubular epithelial cell; PCNA Proliferating cell nuclear antigen; Smurf2 Smad-specific E3 ubiquitin protein ligase 2. Figure created with BioRender.com

TECs exhibit robust self-repair capacity following mild renal injury. However, serious or persistent injury results in maladaptive repair of damaged TECs that perpetuates renal inflammation and fibrosis. These TECs that are inadequately repaired display characteristics such as stagnation in the cell cycle, dysfunctional mitochondria, and altered metabolic processes, which can persist following acute kidney injury. They drive intricate crosstalk with immune populations, particularly macrophages, via inflammatory mediators and extracellular vesicles (EVs), thereby amplifying the intrarenal inflammatory response. Furthermore, maladaptive TECs secrete key profibrotic factors, which collectively drive fibroblast activation and tubulointerstitial fibrosis [21]. Figure 1 summarizes the core cellular events and crosstalk that drive adaptive or maladaptive repair and renal fibrosis. Resetting these persistent cellular abnormalities potentially halts AKI-CKD transition.

### Persistent cell cycle arrest, cellular senescence and polyploidization

Following AKI, surviving TECs compensate for the loss of adjacent cells through dedifferentiation, migration, and proliferation. This repair process relies on stringent cell cycle control, in which successful mitosis generates daughter cells that eventually redifferentiate to restore tubular integrity [22]. During the acute phase, TECs activate self-protective mechanisms, such as cell cycle arrest or polyploidization, to prevent the propagation of damaged genetic information and maintain cell mass [23]. The cell cycle is orchestrated by cyclin-dependent kinase (CDK) complexes and negatively regulated by suppressor proteins, including p53, retinoblastoma protein (Rb), and p21 [24]. However, this balance is remarkably fragile. Prolonged or inappropriate cycle arrest and polyploidization drive TECs toward a senescent and profibrotic phenotype.

G2/M cell cycle arrest in TECs is a prominent mechanism in renal fibrosis. G2/M-arrested TECs activate the c-Jun N-terminal kinase (JNK) and upregulate the transcription of transforming growth factor-β1 (TGF-β1) and connective tissue growth factor (CTGF), thereby promoting fibrosis [25]. P53 inhibits the G1/S transition via p21 while also suppressing the CDK1/cyclin B1 complex by inducing 14-3-3σ and growth arrest and DNA damage-inducible 45 (Gadd45), thereby locking cells in the G2/M phase [26]. Therefore, blocking this process or its downstream signaling effectively suppresses fibrosis progression. Recent studies reveal that circBNC2 enhances CDK1/cyclin B1 complex assembly, thereby alleviating maladaptive repair [27]. Furthermore, Cyclin G1 (CG1) promotes TOR-dependent autophagy-space coupling structures (TASCs) and induces a senescence-associated secretory phenotype (SASP). Inhibiting CG1 or blocking TASC formation significantly alleviates G2/M arrest and fibrosis. Although CDK5 deficiency does not reverse G2/M arrest, it effectively suppresses dedifferentiation and fibrosis, highlighting the pathogenic role of the CG1-CDK5 axis in maladaptive repair [28, 29]. At the single-cell level, TECs with persistent cycle arrest exhibit unique molecular signatures, forming distinct proinflammatory and profibrotic subpopulations, as detailed in the following section.

Oxidative stress and DNA damage following AKI are potent inducers of cellular senescence [30, 31]. Senescent TECs upregulate cyclin-dependent kinase inhibitor 2 A (p16), 1B (p27), and 1 A (p21) and secrete diverse cytokines and growth factors via the SASP. These factors impair adjacent healthy cells, and trigger inflammatory cascades, myofibroblast proliferation, and excessive extracellular matrix (ECM) accumulation, thereby driving renal fibrosis [32]. The mechanisms underlying ischemia–reperfusion injury (IRI)-induced senescence are multifaceted and involve telomere shortening, DNA damage, mitophagy, metabolic dysregulation, endoplasmic reticulum (ER) stress, and epigenetic remodeling [31]. Morphologically, senescent TECs typically appear as enlarged, flattened cells with condensed chromatin and prominent nuclear staining. These cells undergo an irreversible cell cycle arrest, rendering them unable to proliferate or differentiate even under stimulation, which further exacerbates renal dysfunction [33].

Protein disulfide isomerase family A member 3 (PDIA3)-expressing senescent TECs activate the TGF-β signaling pathway post-injury, subsequently driving fibroblast activation and accelerating fibrotic progression [34]. Additionally, a subpopulation of cells exhibiting both epithelial-mesenchymal properties and high expression of senescence-related genes has been identified in the renal artery stenosis model. Administration of senolytic drugs such as AP20187, Dasatinib and Quercetin, alleviated renal function decline by removing these senescent cells [35]. Targeting the reactive oxygen species (ROS)/Sirtuin 1 (SIRT1)/p53 signaling pathway mitigates cellular senescence and markedly reduces fibrosis [36]. Additionally, vanin-1 (VNN1) has emerged as another critical driver, inducing tubular senescence by activating retinoblastoma transcriptional corepressor 1 (RB1) and phosphorylation following severe injury, thus facilitating the AKI-CKD transition [37]. Pannexin 1 (Panx1)-mediated endoplasmic reticulum Ca^2^⁺ leakage is another key driver of kidney disease progression. Panx1 triggers tubular senescence by disrupting mitochondrial homeostasis. Consequently, therapeutic agents targeting Panx1 may represent a strategy for limiting renal senescence and subsequent fibrosis.

DNA damage repair and polyploidization serve as complementary mechanisms to maintain renal function post-injury [38, 39]. Residual differentiated parenchymal cells experience endoreplication hypertrophy, leading to the formation of polyploid cells that sustain renal function [40, 41]. While Yes-associated protein 1 (YAP1)-driven polyploidy initially serves a self-protective role by inducing cortical hypertrophy to preserve residual function, these cells eventually transition toward a senescent phenotype in the later stages, thereby promoting interstitial fibrosis [42]. During CKD progression, polyploid tubular cells accumulate DNA damage and secrete TGF-β1, thereby enhancing crosstalk with macrophages and fibroblasts and further exacerbating fibrosis [42–44]. Another study found that macrophages secrete interferon beta (IFN-β) via activation of the cyclic GMP-AMP synthase (cGAS)- stimulator of interferon genes (STING) pathway. This interferon acts on the tubular surface receptor interferon α/β receptor subunit 1 (IFNAR1), inducing dephosphorylation of YAP in tubular cells. This leads to its nuclear translocation, where it regulates TECs polyploidization [45].

### Chronic inflammation and immune cell infiltration

Under pathological conditions such as infection, IRI, or nephrotoxicity insult, TECs undergoing apoptosis, ferroptosis, necrosis, or pyroptosis as well as TECs in a maladaptive repair state, release intracellular contents and cytokines, thereby initiating inflammatory responses in adjacent cells [21]. Innate immunity is primarily initiated by acute release of damage-associated molecular patterns (DAMPs), such as high-mobility group box 1 (HMGB1), uric acid, and nucleic acid fragments from injured TECs. These signals are recognized by Toll-like receptors (TLRs), particularly TLR2 and TLR4 on TECs and immune cells. The innate immune system normally maintains tissue homeostasis and defends against injury through rapid responses to DAMPs/pathogen-associated molecular patterns (PAMPs). However, excessive activation of this pathway amplifies the inflammatory cascade, ultimately exacerbating parenchymal damage [46]. This mainly occurs as a significant and ongoing penetration of both innate and adaptive immune cells into the kidneys. Immune cells, both resident and infiltrating, extensively interact with endothelial cells, TECs, and pericytes during the inflammatory response.

#### Neutrophils

Neutrophils are among the earliest innate immune cells recruited to the injured kidney during the early stages of AKI [47, 48]. They engage in the inflammatory response via several effector mechanisms, including generation of ROS, degranulation, and release of neutrophil extracellular traps (NETs) [48–51]. NET formation and its proinflammatory effects exacerbate IRI [52, 53]. Conversely, neutrophil depletion or blocking their recruitment significantly attenuates tissue damage [54, 55]. Neutrophil infiltration is intimately connected with inflammation and fibrosis in AKI-CKD transition. During the late phase of injury, macrophages accumulate extensively in the atrophic kidney, recruiting proinflammatory neutrophils and T cells, which is characterized by a distinct secondary inflammatory surge. Depleting neutrophils and T cells after the fifth day following the injury reduces tubular cell loss and mitigates subsequent renal atrophy [56]. Neutrophils lacking epidermal growth factor receptor (EGFR) reduced anti-apoptotic myeloid cell leukemia 1 (MCL-1) levels and increased apoptosis, accelerating recovery from renal injury and diminishing subsequent fibrosis [57]. Gasdermin E (GSDME)-driven TEC death induces NET formation and drives proinflammatory macrophage formation and macrophage-myofibroblast transition (MMT) responses, whereas gasdermin D (GSDMD) deficiency blocks NETs formation and subsequent profibrotic processes [58, 59]. In unilateral ureteral obstruction (UUO) models, sialic acid-binding immunoglobulin-like lectin F (Siglec-F) + neutrophils in injured kidneys exhibit enhanced profibrotic phenotypes. Their accumulation and activation promote fibroblast activation, exacerbating collagen deposition and disease progression, whereas their depletion attenuates renal fibrosis [60]. Conversely, CD300a deficiency in Siglec-F-low neutrophils increases the production of proangiogenic and anti-fibrotic molecules induced by signal transducer and activator of transcription 3 (STAT3), thereby slowing CKD progression.

#### Macrophages

During the acute phase of AKI, proinflammatory macrophages accumulate in the injured area, initiating and amplifying the immune response [61–63]. Chemokine C-X-C motif ligand 2 (Cxcl2) + macrophages exhibit high expression of proinflammatory genes and chemokines, including interleukin-1β (IL-1β), tumor necrosis factor (TNF), Cxcl3, and chemokine CC motif receptor-like 2 (Ccrl2), and persist even in the late stage of injury. This suggests a potential role in activating and sustaining the inflammatory response [61, 62]. In contrast, resident or mannose receptor 1 (Mrc1) + and matrix metalloproteinase-12 (Mmp12) + macrophages are more likely to promote repair through self-renewal and are crucial in sustaining homeostasis after injury [62–67]. Research indicates that kidney resident macrophages (KRMs) comprise seven distinct subpopulations with specific spatial organization. Renal injury disrupts this original niche structure, leading to imbalance even 28 days post-injury, which contributes to the kidney’s inability to restore homeostasis [68].

In the chronic stage, profibrotic and proinflammatory macrophage subsets increasingly outnumber reparative populations. A recently identified monocyte-derived macrophage subset participates in transient matrix remodeling before transitioning to a profibrotic phenotype. These cells directly drive fibrosis progression by establishing complex ligand-receptor interactions with fibroblasts [62]. Furthermore, macrophage inducible C-type lectin (Mincle) + macrophages provide sustained inflammatory momentum for the AKI-CKD transition by promoting tumor necrosis factor-alpha (TNF-α) production [48]. Recent studies indicate that macrophages switch to a profibrotic phenotype driven by platelets and other factors. Platelet-derived Cxcl4 promotes post-AKI fibrogenesis by driving the differentiation of profibrotic macrophage subpopulations expressing secreted phosphoprotein 1 (SPP1), fibronectin 1 (FN1), and arginase 1 (Arg1). This process activates fibroblasts through the SPP1/FN1/semaphorin 3 (Sema3) signaling axis [69]. Similarly, platelet-derived thrombospondin 1 (THBS1) stimulates the differentiation of macrophages into a distinct and highly proliferative M2-like subtype that exacerbates kidney fibrosis by prompting excessive production of extracellular matrix components [70]. Beyond intra-renal factors, the gut-derived metabolite trimethylamine N-oxide (TMAO) has been shown to enhance Ccr2 + macrophage infiltration, thereby accelerating AKI-CKD progression [65]. The potent crosstalk between macrophages and neutrophils, mediated by C–C motif chemokine ligand 9/6(Ccl9/6)-Ccr1 signaling, further drives innate immune activation in the UUO model [71]. Therapeutic interventions targeting macrophage and other immune cell recruitment, or modulating the behavior of intrinsic renal cells, may represent promising strategies to prevent AKI-CKD transition.

#### T lymphocytes and innate lymphoid cells

While the initial inflammatory response in AKI was traditionally attributed to innate immunity, accumulating evidence from IRI and cisplatin-induced models has redefined the role of T cells. These cells rapidly infiltrate the injured kidney during the hyperacute phase, serving as critical amplifiers of the inflammatory cascade [46, 72, 73]. Deficiency of CD4, CD8, or T cell receptor significantly attenuates IRI [74, 75]. Different CD4 + T cell subsets exhibit distinct functional roles in AKI. T helper 1 (Th1) cells exacerbate injury by mediating proinflammatory effects via interferon-γ (IFN-γ) and TNF, whereas Th2 cells partially limit inflammation and exert protective effects [76, 77]. Th17 cells undergo transient expansion in ischemic AKI and promote inflammation through IL-17. Their pathogenicity is regulated by galectin-8 and ORAI calcium release-activated calcium channel protein 1 (Orai1)-dependent calcium signaling [78, 79]. In contrast, double-negative T cells exhibit anti-inflammatory properties [80].

Moderate to severe ischemic injury results in the prolonged retention of activated and effector memory CD4 + and CD8 + T cells, accompanied by sustained upregulation of proinflammatory factors such as IL-1β, IFN-γ, and CCL5. This sustained adaptive immune activation, if left unresolved, serves as a crucial factor in AKI-CKD transition [81, 82]. Notably, blocking CCR5, which is upregulated in renal infiltrating T cells not only mitigates renal dysfunction and histological alterations post-IRI but also markedly suppresses T cell activation within the kidney [83, 84]. Recent single-cell transcriptomic sequencing (scRNA-seq) studies reveal that sustained crosstalk between injured TECs and renal macrophages establishes a chronic inflammatory niche that drives a secondary wave of immune activation. Activated macrophages promote CD8 + T cell recruitment via the CXCL16-C-X-C motif chemokine receptor 6 (CXCR6) axis, and these pathogenic T cells subsequently induce endothelial apoptosis through Fas ligand (FasL)-Fas receptor (Fas) signaling, resulting in peritubular capillary rarefaction and progressive interstitial fibrosis [85]. In parallel, chemokine-high macrophage subsets expressing CXCL16 and CCL8 orchestrate a second wave of immune infiltration, characterized by CCR1 + neutrophils, macrophages, and CXCR6 + T cells, which damaged TECs again. Targeted depletion of T cells and neutrophils attenuates this maladaptive immune activation, mitigates tubular injury and atrophy, and limits fibrosis progression, underscoring a central role for macrophage-driven T cell responses in perpetuating chronic inflammation [56].

Regulatory T cells (Tregs) are recognized as a crucial immune population that promotes tissue repair and persist into the late fibrotic stage of AKI. They highly express genes associated with anti-inflammation and tissue repair and release TGF-β1 and IL-10 to suppress the production of inflammatory factors [86]. Suppression of tumorigenicity 2 (ST2)-expressing Tregs coordinate renal repair in the late phase of injury by secreting amphiregulin (AREG) [87], while early activation of the IL-33-ST2 signaling axis induces renal Treg expansion, thereby mitigating acute injury and limiting fibrosis progression [88]. However, Treg function is highly dependent on the immune microenvironment. Recent studies indicate that in the absence of NKT cells, Tregs accumulate excessively with enhanced TGF-β expression, creating a pro-fibrotic immune environment that accelerates renal fibrosis progression. This suggests natural killer T (NKT) cells play a critical protective role in limiting pathological fibrosis in CKD by finely regulating Treg numbers and functional states. Innate lymphoid cells (ILCs) constitute a minor yet functionally diverse immune compartment in the kidney, comprising ILC1s, ILC2s, and ILC3s, which mirror Th1-, Th2-, and Th17-like programs [89]. Experimental evidence indicates that ILC2s suppress inflammation and promote renal repair during AKI by secreting IL-5 and IL-13, thereby driving M2 macrophage polarization [90, 91]. However, sustained IL-33 stimulation results in persistent ILC2 activation, excessive IL-13 production, and prolonged M2 polarization, ultimately favoring fibrogenesis. Progressive renal injury leads to excessive and prolonged IL-33 release, thereby exacerbating kidney damage and fibrosis [92]. In parallel, ILC3s have emerged as a distinct profibrotic subset, rapidly trafficking from the gut to the injured kidney via the CXCR6-CXCL16 axis, where they directly activate fibroblasts and accelerate kidney fibrosis [93], forming a crucial bridge linking the gut-kidney axis to fibrosis.

#### Other immunological factors

Dendritic cells (DCs) serve as pivotal nodes linking innate and adaptive immunity. Dendritic cells in immature state undergo a transformation in phenotype and maturation when exposed to alarmins including granulocyte–macrophage colony-stimulating factor (GM-CSF) and IL-33, subsequently promoting T cell activation and proliferation through efficient antigen presentation [94, 95]. Current research indicates DCs exhibit high heterogeneity in renal injury and fibrosis [67, 96], exerting both pro-inflammatory and pro-reparative effects. The cDC2 subset exacerbates transplant injury and IRI by secreting pro-inflammatory factors. cDC1s promote tissue repair in AKI and UUO models by directly releasing IL-22 or inducing Tregs to produce IL-4, IL-10, and other factors [97]. Furthermore, the complement system is critical in the progression of AKI-CKD. Complement C3 is significantly increased and persistently expressed in failed repair proximal tubular cells (FR-PTCs), driving AKI-CKD transition through complement-mediated crosstalk with immune cells and matrix components [98]. The complement activation product complement component 5a (C5a) accelerates pathological progression by inducing aberrant methylation in TECs, stimulating Wnt/β-catenin signaling, and promoting SASP [99].

### Sustained fibroblast activation and extracellular matrix deposition

Renal fibrosis development hinges on myofibroblast activation. Uncontrolled ECM accumulation during this process causes irreversible scarring in the kidney [100–102]. Recent single-cell data have redefined fibroblasts and myofibroblasts as highly heterogeneous populations in fibrotic kidneys. While the most abundant subpopulation continues to drive classical fibrogenesis through ECM-related gene expression, specialized subsets underscore the functional complexity of the fibrotic kidney [63, 103]. Studies have identified contractile fibroblast subpopulations enriched in myogenic genes and migratory subpopulations enriched for immunoregulatory genes [103]. Of particular note is a transitional inflammatory fibroblast subpopulation termed CXCL-iFibro, observed in CKD patients. These transitional fibroblasts accumulate early in the injured kidney and can recruit and activate folate receptor beta (FOLR2) + macrophages. Notably, macrophages further promote the transition of CXCL-iFibro into ECM-secreting myofibroblasts through Wnt/β-catenin-dependent signaling pathways [104], constituting a fibroblast-macrophage profibrotic circuit. In addition, a unique myofibroblast subset marked by high levels of IL-34 and myosin, alongside activated extracellular signal-regulated kinase (ERK)/mitogen-activated protein kinase (MAPK) signaling cascades, has been implicated in the sustained progression and persistence of renal inflammatory responses [63].

Tissue damage induces fibroblast activation through soluble molecules, EVs, matrix proteins, and mechanical signals derived from TECs and macrophages. Together with inflammatory immune cells and metabolites, these factors orchestrate a profibrotic niche that drives ECM remodeling [105, 106]. Damaged or inflamed TECs serve as a major source of signals for fibroblast activation in AKI-CKD transition. They promote fibroblast proliferation, activation, and myofibroblast differentiation through multiple ligand-receptor axes [63, 107, 108]. The inflammation-associated epithelial subpopulation, iPT, secretes Indian Hedgehog in response to TNF and nuclear factor-κB (NF-κB) activation, promoting the expansion of glioma-associated oncogene 1 (Gli1) + pericytes and fibroblasts, thereby amplifying fibrotic responses in the kidney and even the heart [109]. Spatial transcriptomics reveals that epithelial-mesenchymal communication exhibits pronounced distance dependence, with fibroblasts near damaged tubules receiving stronger signals from cardiotrophin-like cytokine factor 1 (CLCF1)-cytokine receptor-like factor 1 (CRLF1), Jagged1 (JAG1)-notch receptor 3 (Notch3), vascular cell adhesion molecule 1 (VCAM1)/SPP1-integrin, and CD44-fibroblast growth factor receptor 2 (FGFR2) pathways [110]. Spatial transcriptomics further reveals that tenascin-C (TNC) is highly enriched in colocalized regions of fibroblasts and macrophages, positioning it as a key ECM molecule in the construction of the fibrogenic niche. Primarily secreted by activated fibroblasts, TNC promotes macrophage recruitment and activation, forming a positive regulatory circuit that amplifies inflammation and ECM deposition [111]. Targeting myofibroblast differentiation or specific intracellular signaling pathways within myofibroblasts offers a promising avenue for treating kidney fibrosis [112, 113].

### Capillary rarefaction and chronic hypoxia

The renal vasculature, a complex network of glomerular and peritubular capillaries, arterioles, and venules, is indispensable for maintaining renal perfusion and tissue homeostasis [114]. In models of renal IRI, the combined effects of vasoconstriction, edema in tissues, swelling of endothelial cells, and capillary breakdown result in the rarefaction of peritubular capillaries and a notable decrease in local blood flow. The resulting persistent hypoxia within the tubular microenvironment disrupts repair and promotes interstitial fibrosis [115]. This vascular collapse is often exacerbated by reduced secretion of vascular endothelial growth factor A (VEGF-A) from injured TECs which impairs endothelial regeneration and further diminishes capillary density [116].

Maladaptive vascular repair, characterized by impaired angiogenesis, chronic hypoxia, capillary rarefaction, endothelial-mesenchymal transition (EndMT), and pathological vascular remodeling, has become a key factor in CKD progression [116–119]. ScRNA-seq has uncovered pronounced endothelial heterogeneity in kidney injury, identifying aberrantly activated endothelial subsets, such as Rap guanine nucleotide exchange factor 5 (Rapgef5) + and membrane-associated guanylate kinase, WW and PDZ domain containing 1 (Magi1) + cells, that are associated with defective angiogenesis and sustained endothelial activation [63]. Moreover, loss of endothelial integrity is accompanied by a reduction in endothelial cell numbers and the acquisition of fibroblast-like transcriptional programs, including upregulation of vimentin (VIM), S100 calcium-binding protein a4 (S100A4), and EndMT-related genes, directly contributing to fibrotic progression [120].

Mechanistic studies demonstrate that preserving or restoring endothelial regenerative capacity counteracts these maladaptive processes. Experimental modulation of endothelial survival, angiogenic signaling and metabolic state reveals that intact tyrosine kinase receptor (Tie2) signaling, glycolytic competence of peritubular capillary endothelial cells, and sustained VEGF-A-vascular endothelial growth factor receptor 2 (VEGFR2) communication from injured TECs are all required to maintain endothelial identity, prevent EndMT and limit microvascular rarefaction [121]. Collectively, these findings suggest that endothelial dysfunction, together with microvascular loss, constitutes a central pathogenic link between AKI and CKD. Hepta-ANG1 therapy alleviates renal injury by promoting the repair of Tie2-dependent endothelial cell populations involved in glomerular and capillary development. Overexpression of 6-phosphofructokinase/fructose-2,6-bisphosphatase 3 (Pfkfb3) in peritubular endothelial cells reverses glycolytic suppression induced by oxidative stress, promotes cell proliferation, and mitigates microvascular sparsity and renal fibrosis [119]. Intermedin prevents the uncoupling of endothelial nitric oxide synthase (eNOS) and reduces oxidative stress through the activation of the AMP-activated protein kinase (AMPK)/GTP cyclohydrolase 1 (GTPCH-I)/tetrahydrobipterin (BH4) signaling pathway, thereby maintaining capillary density and reducing renal fibrosis. This provides a new therapeutic approach by addressing endothelial dysfunction [122]. Recent studies indicate that overexpression of syndecan 1 (SDC1) in renal TECs after moderate AKI enhances the release of VEGF-A, promoting endothelial proliferation and vascular repair by activating endothelial VEGFR2 signaling. Collectively, these studies emphasize that endothelial dysfunction and impaired vascular repair constitute the core pathological link connecting AKI to CKD. Meanwhile, a deficiency in eNOS leads to endothelial dysfunction, which in turn hastens AKI-CKD transition by facilitating prolonged activation of β-catenin in macrophages [123].

### Impaired tubular cellular regeneration and fate misregulation

The resolution of proximal tubular injury ultimately hinges on a critical bifurcation of cellular fate, either the pursuit of functional regeneration through an orchestrated cell cycle or a descent into a maladaptive, pro-fibrotic phenotype. Recent scRNA-seq studies have precisely delineated these FR-PTCs. These cells undergo G2/M phase arrest and senescence, representing core pathological drivers of AKI-CKD transition [124]. Evidence suggests that senescent proximal TECs, when persistently trapped in a state of arrest, exhibit a robust molecular signature characterized by the high expression of VCAM1 [84, 103, 125–127]. This VCAM1 + population, virtually absent in the healthy kidney, expands significantly post-injury to become the definitive hallmark of failed repair [128]. Mechanistically, these cells transduce complex molecular signals, such as aberrant activation of the NF-κB and activator protein 1 (AP-1) pathways, into sustained proinflammatory and profibrotic phenotypes. By secreting SASP factors such as CCL2, they recruit and activate immune cells and fibroblasts within the stroma [126, 127]. Abnormal expression of transcription factors, particularly nuclear factor of activated T-cells 5 (NFAT5), Krüppel-like factor 6 (KLF6), and runt-related transcription factor 1 (RUNX1), sustains the dysregulated state of this cellular subset [125, 129–131].

Unlike VCAM1 + cells that progress toward maladaptive responses, a subset of proximal TECs attempts to reverse injury by reacquiring developmental plasticity. Activation of the sex-determining region Y box 9, commonly known as SOX9, marks the initiation of this adaptive regenerative process [132, 133]. These SOX9 + cells represent an endogenous repair pool within the kidney that enters a proliferative cycle in response to upstream signaling, such as the epidermal growth factor receptor (EGFR)-protein kinase B (AKT)-YAP pathway, to restore tubular integrity through epithelial regeneration [132]. Beyond their own proliferation, endogenous SOX9 + TECs can activate neighboring cells. This is achieved through the autocrine and paracrine secretion of molecules including S100 calcium-binding protein A9 (S100A9), underscoring their fundamental role in orchestrating kidney repair [134]. However, this reparative potential is highly time-sensitive. Successful regeneration depends on timely activation of SOX9 signaling followed by its active silencing. When cells exhibit persistent SOX9 expression due to severe injury or a deteriorating local environment, they may engage in abnormal signaling exchanges with mesenchymal fibroblasts via the Wnt pathway and accelerated fibrosis.

Besides proximal TECs dedifferentiation, it is essential to activate endogenous renal progenitors to alleviate tubular atrophy caused by injury. Emerging data suggest that leucine-rich repeats and immunoglobulin-like domains protein 1 (LRIG1) + cell populations function as progenitors to drive structural recovery post-AKI [135]. Scattered tubular cells (STCs) represent a reversible dedifferentiated state, though impaired redifferentiation leads to a sustained proinflammatory secretory profile [136, 137]. Under severe injury conditions, Wilms’ tumor 1 (WT1) + glomerular parietal epithelial cells can be recruited to transdifferentiate into proximal TECs via TGF-β- and Notch-dependent pathways [138]. These diverse endogenous repair mechanisms, along with their associated signaling networks, form a critical framework for renal regeneration and provide specific targets for promoting repair.

## Key molecular signaling pathways in the AKI-CKD transition

The AKI-CKD transition represents a multifaceted pathological phenomenon driven by persistent renal inflammation, tubular dysfunction, and excessive ECM deposition, which ultimately leads to irreversible renal fibrosis and ESRD. Accumulating evidence has demonstrated that the dysregulation of several pivotal signal axes significantly contributes to this transition. These pathways orchestrate the crosstalk between renal TECs, fibroblasts, macrophages, and other renal resident cells, thereby governing cellular fate, inflammatory responses, metabolic homeostasis, and epigenetic remodeling [139–141]. Below, we systematically elaborate on the roles and regulatory mechanisms of several core signaling pathways, including TGF-β/suppressor of mothers against decapentaplegic homolog (Smad), Wnt/β-catenin, Hedgehog, NF-κB/NOD-like receptor family pyrin domain-containing 3 (NLRP3) inflammasome, as well as metabolic reprogramming and epigenetic regulation in the AKI-CKD transition, seeking to deliver an in-depth insight into the molecular mechanisms that govern this process and to propose possible therapeutic targets for clinical intervention.

### TGF-β/Smad signaling

Damaged renal TECs significantly upregulate and secrete TGF-β1 following AKI [25, 43]. Upon binding to TGF-β type I and II receptors (TGFβRI/II), TGF-β1 activates the Smad2/3 signaling cascade. Phosphorylated Smad2/3 forms a heteromeric complex with Smad4, which then translocates into the nucleus to directly initiate the transcription of profibrotic genes [142]. This process induces epithelial-mesenchymal transition (EMT) and fibroblast-to-myofibroblast transition, driving progressive renal interstitial fibrosis [143]. Recent research has further revealed homeobox C8 (Hoxc8) as a critical downstream effector that interacts with the positive transcription elongation factor b (P-TEFb) to amplify the expression of fibrotic genes [144]. Smad7 functions as an endogenous negative regulator by inhibiting TGF-β receptor signaling to limit Smad3 activity under physiological conditions. However, under sustained TGF-β1 stimulation, Smad7 is frequently degraded, leading to failure of the anti-fibrotic braking mechanism [145, 146].

**Fig. 2:**
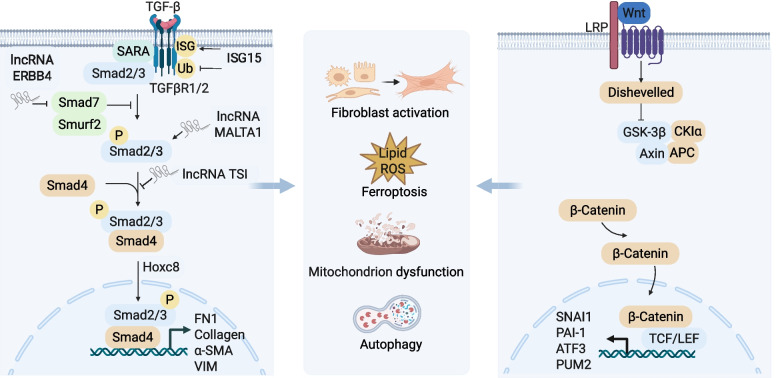
The role of TGF-β/Smad and Wnt/β-Catenin pathways in AKI-CKD progression. TGF-β activates Smad2/3-Smad4 complex nuclear translocation to promote fibrosis-related gene expression. Molecules such as IncRNA ERBB4-IR, MALAT1, TSI modulate this pathway by targeting different mediator. Wnt signaling inhibits β-Catenin degradation, enabling its nuclear translocation to drive transcription of pro-fibrotic genes such as SNAI1 and PAI-1. Together, they induce fibroblast activation, ferroptosis, and mitochondrial dysfunction, promoting renal fibrosis and AKI-CKD transition. α-SMA α-smooth muscle actin; APC adenomatous polyposis coli protein; ATF3 activating transcription factor 3; AXIN axin protein; CK1α casein kinase 1α; FN1 fibronectin 1; GSK-3β glycogen synthase kinase 3β; Hoxc8 homeobox C8; lncRNA long non-coding RNA; ISG15 interferon-stimulated gene 15; LRP low-density lipoprotein receptor-related protein; PAI-1 plasminogen activator inhibitor 1; PUM2 pumilio RNA binding family member 2; ROS reactive oxygen species; Smad suppressor of mothers against decapentaplegic homologue; SNAI1 snail family transcriptional repressor 1; Smad2/3/4/7 mothers against decapentaplegic homolog 2/3/4/7; TCF T cell factor; LEF lymphoid enhancer factor; TGF-β transforming growth factor-β; TGFβR1/2 transforming growth factor-β receptor type 1/2; VIM vimentin; SARA Smad anchor for receptor activation. Figure created with BioRender.com

The TGF-β signaling pathway is extensively activated in AKI-CKD transition and its activity and function are also finely regulated at multiple levels and through diverse mechanisms. Figure 2 provides a schematic summary of the multi-level regulation and functional effects mediated by TGF-β signaling in AKI-CKD transition. Interferon-stimulated gene 15 (ISG15) was verified to modify TGFβR1 via ISGylation, suppress its ubiquitin-mediated degradation, sustain TGF-β pathway activation, and aggravate renal fibrosis [147]. Besides, long non-coding RNAs (lncRNAs) modulate of the TGF-β/Smad signaling axis to govern fibrotic progression. Specific lncRNAs such as ERBB4 intronic region (ERBB4-IR) and metastasis-associated lung adenocarcinoma transcript 1 (MALAT1) deficiency promote fibrosis by stabilizing Smad signaling or inducing EMT [148, 149]. Conversely, lnc-TSI exerts a protective effect by binding to the MH2 domain of Smad3 to inhibit the downstream fibrogenic cascades [150]. Inactivation of serum/glucocorticoid-regulated Kinase 3 (SGK3) within TECs exacerbates EMT and increases TGF-β1 secretion by downregulating T-LAK cell-originated protein kinase (TOPK) phosphorylation. TGF-β1 then acts on macrophages, inducing their transformation toward a profibrotic phenotype and MMT [151]. Notably, in addition to directly regulating matrix synthesis, TGF-β1 participates in novel cell death mechanisms. Smad3 directly binds the glutathione peroxidase 4 (GPX4) promoter to suppress its expression, thereby inducing ferroptosis and promoting renal fibrosis progression [152]. Additionally, TGF-β1 mediates myofibroblast activation, excessive ECM production, and degradation inhibition through non-canonical pathways such as MAPKs and phosphoinositide 3-kinase (PI3K)/AKT. The rapid activation of TGF-β/Smad signaling during the acute phase of AKI and its positive correlation with injury severity make it a crucial indicator for predicting CKD outcomes. Consequently, focusing on this pathway and its crucial components substantially reduces renal fibrosis in AKI-CKD transition, while also enhancing renal function, thus highlighting its considerable therapeutic potential [147, 153].

### Wnt/β-catenin signaling

The Wnt/β-catenin pathway is critical for kidney development but typically remains quiescent in the adult kidney [154]. After the onset of AKI, transient activation of this pathway decreases apoptosis and promotes the proliferation of proximal TECs, along with tissue repair and cellular regeneration [155, 156]. However, sustained Wnt signaling constitutes a key hallmark of AKI-CKD transition [154]. As shown in Fig. 2, cytoplasmically accumulated β-catenin translocate to the nucleus, where it binds to T cell factor (TCF)/lymphoid enhancer factor (LEF) transcription factors to upregulate snail family transcriptional repressor 1 (SNAI1), Plasminogen activator inhibitor 1 (PAI-1), and various myofibrillar genes. This induces an EMT-like phenotypic shift in TECs, thereby initiating and sustaining the fibrotic process. Besides, overexpression of Wnt family member 5 A (WNT5A) in proximal TECs promotes CD146 upregulation, activates JNK-mediated phosphorylation of Jun proto-oncogene (c-JUN), enhances its interaction with KLF5 at the SNAI1 promoter, and ultimately drives renal fibrosis [157]. Meanwhile, activation of Wnt/β-catenin/activating transcription factor 3 (ATF3) signaling promotes AKI-CKD transition by regulating mitochondrial dynamics and mitophagy [158]. Wnt/β-catenin suppresses YME1-like mitochondrial metalloprotease (YME1L)-mediated mitochondrial homeostasis by activating Pumilio RNA binding family member 2 (PUM2) transcription, exacerbating renal fibrosis [159]. Inhibitor of nuclear factor-B kinase subunit α (IKKα) overexpression intensifies renal fibrosis by activating the Wnt/β-catenin pathway through enhanced β-catenin nuclear translocation [160].

### Hedgehog signaling

**Fig. 3:**
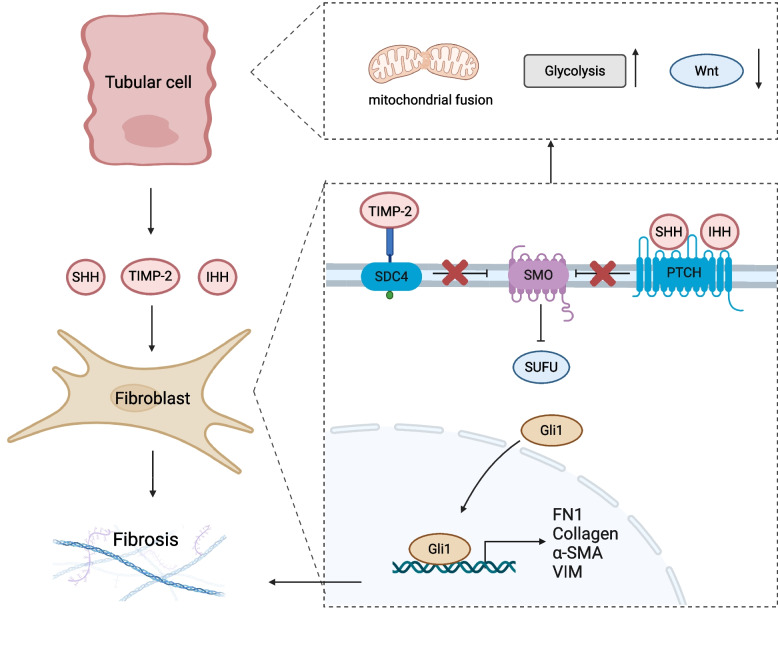
The Hedgehog signaling pathway acts as a primary driver of renal fibrosis in AKI-CKD transition. Following injury, TECs secrete ligands such as SHH and IHH alongside TIMP-2 to target renal interstitial fibroblasts. These mediators bind to the PTCH, relieving the inhibition of Smoothened and subsequent SUFU-mediated repression of Gli1. Upon nuclear translocation, Gli1 directly activates pro-fibrotic gene transcription, driving fibroblast activation and excessive ECM deposition. Furthermore, activation of the Hedgehog pathway induces mitochondrial fusion, upregulates glycolysis, and inhibits Wnt signaling in TECs to accelerates AKI-CKD transition. Gli1 Glioma-associated oncogene 1; IHH Indian hedgehog; SHH Sonic hedgehog; SDC4 syndecan 4; SMO smoothened; SUFU suppressor of fused homolog; TIMP-2 tissue inhibitor of metalloproteinase 2; PTCH patched; Figure created with BioRender.com

Hedgehog ligands, as a key class of paracrine signaling molecules, mediate intercellular communication spanning hundreds of micrometers and mediate epithelial-mesenchymal dialogue following renal injury. In vertebrates, this family comprises three ligands including Sonic Hedgehog (SHH), Indian Hedgehog (IHH), and Desert Hedgehog (DHH). When these ligands attach to the membrane receptor Patched receptor (PTCH), these ligands relieve inhibitory effects of Smoothened receptor (SMO) protein, thereby activating downstream signaling pathways. During the renal injury response, Hedgehog signaling pathway regulates AKI-CKD transition through multiple molecular mechanisms as shown in Fig. 3. SHH secreted by TECs acts on Gli1 + fibroblasts in the renal interstitium, driving their proliferation and expression of α-smooth muscle actin (α-SMA), type I collagen, and FN1, thereby directly triggering a profibrotic program that leads to myofibroblastic phenotype conversion [161, 162]. Following AKI, SMO deficiency in Hh-responsive fibroblasts reduces TECs apoptosis and inflammation infiltration while activating Gli1-/Platelet-derived growth factor receptor-β (Pdgfrβ)- fibroblasts, pericytes, and mesenchymal stromal cells [163]. Mechanistically, inhibiting SMO in fibroblasts releases Nidogen 1 (NID1), which activates Wnt signaling in TECs via integrin β1, thereby mitigating AKI [163].

Recent scRNA-seq identified an inflammatory proximal tubule cell (iPT). This cell group secretes IHH upon TNF-α and NF-κB induction, which activates canonical Hedgehog signaling in Gli1 + cells, thereby mediating fibroblast proliferation in injured or aged kidneys. This mechanism is also observed in cardiac fibrosis within the cardiorenal syndrome, suggesting a potential role for IHH in cross-organ fibrosis[109]. Tissue inhibitor of metalloproteinase-2 (TIMP-2) binds to the extracellular region of syndecan 4 (SDC4) via an autocrine pattern, thereby triggering Hedgehog signaling activation. Ciclosporin provided partial protection against mitochondrial dysfunction caused by the overexpression of TIMP-2, suppressed glycolysis mediated by Pfkfb3, and decreased collagen accumulation[164]. Given that the Hedgehog pathway often evades conventional anti-inflammatory therapies, it has become a key focus for efforts aimed at intervening AKI-CKD transition. Studies indicate that IHH deletion, pharmacological inhibition of the SMO receptor, or pharmacological blockade of TNF, NF-κB, or Gli1 signaling significantly attenuates renal fibrosis in injury models, offering novel strategies for clinically interrupting chronic progression.

### NF-κB signaling and the NLRP3 inflammasome

**Fig. 4:**
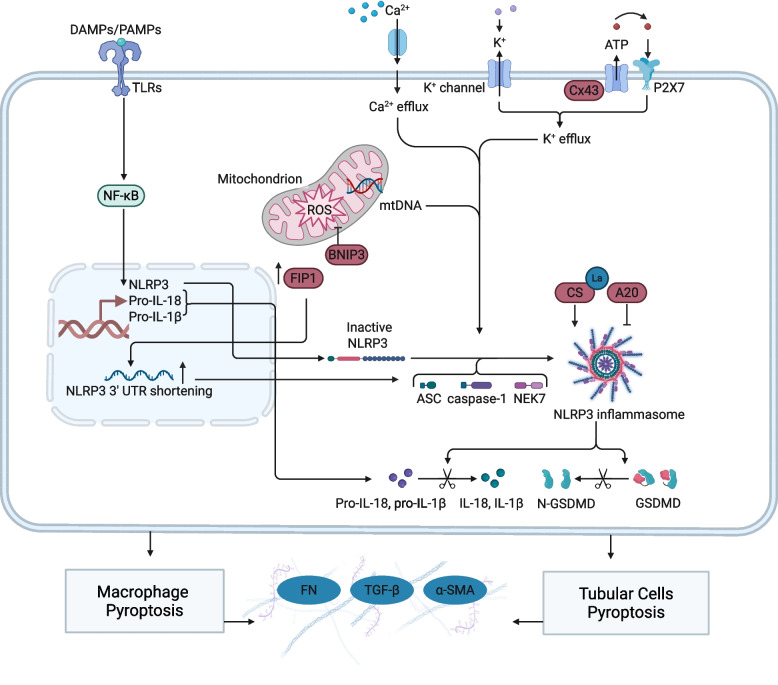
Molecular mechanisms of the NLRP3 inflammasome and NF-κB signaling pathways orchestrate pyroptosis and renal fibrosis in AKI-CKD transition. DAMPs and PAMPs activate the NF-κB pathway via TLRs to upregulate NLRP3 and pro-inflammatory precursors following injury. Concurrently, ionic fluxes and mitochondrial ROS drive the assembly of the NLRP3 inflammasome, which activates caspase-1 to induce GSDMD-mediated pyroptosis in macrophages and TECs. The resulting release of mature IL-1β, and IL-18 triggers fibroblast activation and ECM deposition, ultimately driving renal fibrosis and accelerating the progression to CKD. Molecules including BNIP3, FIP1, CS, A20, and NEK7 regulate of this pathological process by modulating the assembly and activation of the NLRP3 inflammasome. ASC apoptosis-associated speck-like protein containing a CARD; ATP adenosine triphosphate; BNIP3 BCL2 interacting protein 3; Cx43 connexin 43; DAMPs damage-associated molecular patterns; FIP1 factor interacting with PAPOLA and CPSF1; GSDMD gasdermin D; IL-1β interleukin 1β; IL-18 interleukin 18; mtDNA mitochondrial DNA; NEK7 NIMA-related kinase 7; NLRP3 NOD-like receptor family pyrin domain-containing 3; NF-κB nuclear factor kappa-light-chain-enhancer of activated B cells; PAMPs pathogen-associated molecular patterns; P2X7 P2X purinoceptor 7; ROS reactive oxygen species; TGF-β transforming growth factor β; TLR Toll-like receptor; UTR untranslated region; CS citrate synthase. Figure created with BioRender.com

Chronic inflammation serves as the core driver of declining renal function following AKI and progression to CKD. The inflammatory NF-κB pathway is rapidly activated following renal injury. It induces the release of proinflammatory cytokines, promotes the upregulation of NLRP3 and caspase-1, and provides the initial signal for NLRP3 inflammasome activation [165, 166]. Subsequent activation signals, including ROS, K^+^ efflux, and mitochondrial DNA (mtDNA) release, initiate the formation of the NLRP3 inflammasome. This initiates a caspase-1/11 cascade that matures IL-1β and IL-18 while cleaving GSDMD to form membrane pores, ultimately leading to pyroptosis [167]. This persistent inflammatory microenvironment, driven by pyroptosis in TECs, not only causes TECs loss but also recruits massive immune cell infiltration, establishing a pathogenic niche for renal fibrosis [168] (Fig. 4).

NLRP3 is significantly expressed in renal tissue of AKI and CKD patients, suggesting that persistent NLRP3 activation contributes to both acute and chronic kidney damage [169]. In IR-induced AKI models, the mitochondrial ROS (mROS)-thioredoxin-interacting protein (TXNIP)-NLRP3 pathway mediates NLRP3 inflammasomes activation and thus participates in the pathogenesis and progression of AKI [170]. Meanwhile, adenine-fed and UUO renal fibrosis models further confirm that oxidative stress, NLRP3 inflammasome activation, and renal fibrosis are all elevated. NLRP3 is primarily localized in maladaptive repair tubules, which are surrounded by obvious pathological changes such as inflammatory infiltration and fibrotic encapsulation [169]. Persistent abnormal activation of NLRP3 participates in acute renal inflammatory injury and correlates with chronic pathological fibrosis [169]. Conversely, knockout of key components of the inflammasome, such as NLRP3 and Caspase-1, can effectively protect TECs, enhance cell viability, and thereby alleviate the severity of AKI by promoting mitophagy and mitigating mitochondrial damage as well as lipid peroxidation [171, 172].

Recent mechanistic studies have revealed a multidimensional regulatory network governing NLRP3 activation in TECs and macrophage, which is extensively involved in the occurrence and progression of AKI as summarized in Fig. 4. Following AKI induction, significantly elevated lactylation levels of the rate-limiting enzyme citrate synthase (CS) promote tubular injury by activating the NLRP3 inflammasome [173]. Abnormal connexin 43 (Cx43) hemichannel activity in TECs induces inflammation by activating NLRP3 inflammasomes and downstream paracrine signaling [174]. Conversely, hypoxia-inducible factor 1α (HIF-1α)-BCL2 interacting protein 3 (BNIP3)-mediated mitophagy protects against hypoxia-induced renal epithelial injury and fibrosis by reducing mitochondrial ROS and inhibiting NLRP3 inflammasome activation [175]. Furthermore, factor interacting with poly(A) polymerase 1 (FIP1) mediated 3’UTR truncation amplifies proinflammatory effects of NLRP3 by enhancing mRNA stability [176]. Beyond TECs, NLRP3 also exerts pathological effects in macrophages [177]. Macrophage surface TLRs recognize DAMPs released by necrotic renal TECs, assemble the NLRP3 inflammasome, and further activate the proinflammatory properties of macrophages [178]. By interacting with the Lys140 residue of NIMA-related kinase 7 (NEK7), A20 and its derivative peptides hinder the NEK7-NLRP3 interaction, suppress macrophage STING signaling and NLRP3-mediated pyroptosis, and thereby effectively relieve auto-DNA-induced AKI [179]. Notably, NLRP3 deficiency in renal TECs suppresses α-SMA and MMP9 expression and EMT progression independently of caspase-1, IL-1β, or IL-18, revealing a direct pro-fibrotic effect of NLRP3 independent of classical inflammatory pathways [180].

### Metabolic reprogramming and epigenetic regulation

**Fig. 5:**
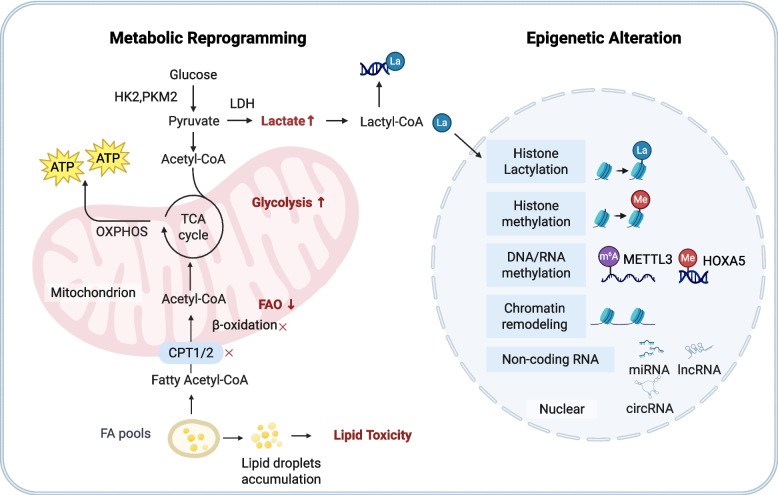
Regulation of metabolic reprogramming and epigenetic alterations during the AKI- CKD transition. TECs undergo enhanced glycolysis alongside suppressed FAO. This metabolic shift promotes intracellular lipid accumulation and lipid droplet formation leading to lipotoxicity. Pyruvate generated from glycolysis is converted by LDH into lactate and subsequently into lactyl-CoA which initiates epigenetic modifications. Alterations in mitochondrial function also cause imbalances in TCA cycle metabolites that further disrupting cellular energy supply and redox status. Besides, aberrant changes occur in histone and DNA or RNA methylation mediated by factors such as METTL3 as well as in chromatin remodeling and the expression of non-coding RNAs. These epigenetic modifications collectively regulate the expression of injury, inflammation or fibrosis-associated genes to drive the progression of renal disease. ATP adenosine triphosphate; circRNA circular RNA; CPT1/2 carnitine palmitoyltransferase 1/2; FA fatty acid; FAO fatty acid oxidation; HK2 hexokinase 2; lncRNA long non-coding RNA; LDH lactate dehydrogenase; METTL3 methyltransferase-like 3; miRNA microRNA; PKM2 pyruvate kinase M2; TCA cycle tricarboxylic acid cycle. Figure created with BioRender.com

The maintenance of physiological functions in TECs is highly dependent on adenosine triphosphate (ATP) supplied by fatty acid β-oxidation (FAO). Impaired FAO represents a core metabolic feature in AKI-CKD transition [181–183]. Impaired transmembrane fatty acid (FA) transport mediated by carnitine palmitoyltransferase 1/2 (CPT1/2) in damaged TECs, coupled with downregulation or loss of activity in key transcription factors peroxisome proliferator-activated receptor-α (PPAR-α) and mitochondrial biogenesis regulator estrogen-related receptor α (ESRRA), diminishes cellular energy supply. This further leads to pathological cytoplasmic accumulation of medium- and long-chain FAs, inducing lipid toxicity [184–187]. To compensate for the energy deficit, TECs undergo a metabolic shift from FAO toward glycolysis (Fig. 5). At this stage, glycolysis-related enzymes such as hexokinase (HK), pyruvate kinase M2 subunit (PKM2), and lactate dehydrogenase (LDH) are significantly upregulated [184]. However, this Warburg-like shift proves insufficiently compensatory, as sustained high glycolytic flux paradoxically promotes inflammation and subsequent renal fibrosis [184, 188]. Furthermore, during the AKI-CKD transition phase, gluconeogenic function in proximal TECs suffers substantial impairment. Clinical evidence indicates that loss of key gluconeogenic enzymes correlates significantly with mortality and chronicity risk in AKI patients [189]. There is a substantial metabolic heterogeneity among distinct PTC subpopulations, and the degree of this metabolic remodeling often determines cellular fate toward either adaptive repair or chronic fibrosis [130, 186, 190–192].

Fibrosis is driven by metabolic dysregulation as well as persistent epigenetic alterations. Epigenetic regulation, including chromatin remodeling, DNA or RNA methylation, histone modifications, and non-coding RNA regulation, drives partial EMT (pEMT) and renal fibrosis by upregulating proinflammatory and profibrotic cytokine production [193]. Chromatin accessibility alterations induced during early injury establish persistent epigenetic memory lasting up to six weeks post-injury [125]. Assay for transposase-accessible chromatin using sequencing (ATAC-seq) analysis revealed Retinoid X receptor alpha (RXRα) structuring and regulating open chromatin domains in mild IRI. Bexarotene, a selective activator of RXRs, protects TECs from severe kidney injury by increasing RXRα recruitment and RXRα-associated chromatin remodeling [194]. Metabolic product accumulation further reshapes the epigenetic landscape. Due to impaired gluconeogenesis-mediated lactate consumption and increased lactate production, lactate accumulates extensively during AKI and is converted to lactyl-CoA. This triggers histone or non-histone lactylation mediated by epigenetic enzymes, locking in pro-fibrotic transcriptional programs [195]. Additionally, abnormal DNA methylation is a key driver of fibrosis [196]. Studies reveal that homeobox A5 (HOXA5) expression is downregulated in fibrotic kidneys due to promoter hypermethylation, which induces JAG1 expression and activates the Notch signaling pathway, driving renal fibrosis progression. Application of the DNA methyltransferase inhibitor 5-Aza or targeted intervention of the HOXA5/Jagged1 (JAG1)/Notch axis significantly suppresses fibrosis [197]. In CKD patients, methyltransferase-like 3 (METTL3) expression and mRNA N6-methyladenosine (m6A) modification levels are significantly elevated in kidneys, driving disease progression by activating the cGAS-STING signaling pathway [198]. Meanwhile, the histone methyltransferase enhancer of zeste homolog 2 (EZH2) directly suppresses phosphatase and tensin homolog (PTEN) transcription by mediating H3K27me3 enrichment in AKI-CKD transition. This activates the EGFR/extracellular signal-regulated kinase 1/2 (ERK1/2)/STAT3 signaling axis, inducing pEMT, G2/M arrest, and impaired transport function in TECs. This exacerbates renal dysfunction and fibrosis [199].

## Biomarkers and early identification of AKI-CKD transition

The adverse impacts of AKI extend far beyond the acute phase, manifesting as recurrent episodes, progressive transition to CKD, heightened cardiovascular issues, and increased mortality rates [200]. It is crucial to promptly recognize patients who are at an increased risk for CKD progression to implement suitable interventions designed to prevent or reverse the deterioration of renal function. Multiple biomarkers are released by renal cells following AKI, which act as vital measurable indicators to objectively reflect renal physiological and pathophysiological alterations. Recently, exploration of biomarkers for AKI-CKD transition has evolved from traditional functional indicators to novel molecular markers, supplemented by advanced imaging and omics technologies, providing a multi-dimensional and multi-level approach for early screening, risk stratification, and prognostic evaluation of high-risk patients.

### Traditional biomarkers

SCr and urine output remain primary indicators for diagnosing AKI and risk stratification in clinical practice [2, 201]. SCr is a standardized and accessible biomarker for estimating GFR, while its clinical value is compromised by its delayed elevation following kidney injury. It lacks sufficient sensitivity for mild or subclinical injury and is affected by extrarenal factors like muscle mass, fluid status, and medications [202]. SCr provides limited insight into the extent of structural damage and the potential for functional recovery. Persistent proteinuria and albuminuria are recognized risk factors for CKD and cardiovascular events, which reflecting ongoing abnormalities in glomerular and tubular function and correlates with incomplete recovery. However, proteinuria lacks sensitivity to early tubular injury and cannot reliably reflect the severity of acute structural damage. Although cystatin C offers a muscle-independent alternative to SCr, it is helpful for patients where SCr is inadequate as a marker or eGFR measurement is impractical [203]. However, its estimation of glomerular filtration function is significantly hampered by non-renal determinants, limiting its clinical utility [204–207]. While traditional biomarkers remain indispensable for clinical management, they are insufficient for achieving early, precise identification of high-risk patients in AKI-CKD transition.

### Novel biomarkers

In contrast to conventional functional biomarkers, novel non-functional biomarkers can reflect molecular and cellular alterations in kidney at earlier acute stress stage. These biomarkers exhibit potential for the early identification of AKI, but their sensitivity and clinical utility are limited if applied indiscriminately to all patients. Consensus on threshold concentrations is required before these markers can be effectively applied in clinical practice [208].

#### Tubular injury biomarkers

KIM-1 is a type I transmembrane glycoprotein with significantly upregulated expression in proximal TECs subsequent to AKI [209]. Due to its extracellular proteolytic domain, KIM-1 is readily detectable in urine. It has received food and drug administration (FDA) approval to sensitively evaluate early renal injury in multiple circumstances [210–213]. Plasma NGAL primarily originates from leakage in damaged thick ascending tubules and collecting ducts following ischemic, nephrotoxic, or infectious renal injury [214–217]. NGAL has shown excellent predictive value for early diagnosis of AKI in the intensive care unit (ICU), even preceding changes in SCr [202, 218]. Other candidate urinary injury-associated biomarkers include liver-type fatty acid-binding protein (L-FABP), insulin-like growth factor-binding protein-7 (IGFBP-7), and angiotensinogen. L-FABP expressed both in the liver and proximal TECs [219]. Urinary L-FABP levels reflect proximal TECs damage linked to lipid overload and oxidative stress, and have been demonstrated to correlate closely with cisplatin, nephrotoxins, and IR-induced AKI [220, 221]. Nonetheless, its elevation generally occurs in the initial stage of injury and gradually returns to baseline after the acute phase. Furthermore, it is affected by hepatic sources and systemic metabolic status, limiting its specificity and long-term predictive value across different temporal phases and particular specific clinical situations [221, 222].

#### Tubular stress biomarkers

Cell cycle arrest associated molecules are significant indicators for the early risk assessment of AKI. TECs undergo G1-phase cell cycle arrest in response to ischemic, toxic, or inflammatory stress to mitigate damage and facilitate repair. Tissue inhibitor of metalloproteinases 2 (TIMP-2) and IGFBP-7 function as critical mediators in this process and can be detected in urine [223]. Urinary [TIMP-2] ·[IGFBP-7] levels effectively predict AKI onset, severity, renal recovery, and mortality risk, particularly in critically ill and AKI associated with cardiac surgery patients. In 2014, the U.S. FDA approved the utilization of urinary [TIMP-2] ·[IGFBP-7] to identify specific critically ill patients at risk for developing moderate to severe AKI [224]. Current research suggests that the clinical relevance of IGFBP-7 primarily relates to short-term risk assessment. Evidence of its value to predict progression of CKD of different etiologies is insufficient and requires further validation through longitudinal investigations. Urinary Dickkopf-3 (uDKK3), a profibrotic glycoprotein derived from TECs, shows elevated levels associated with rapid GFR decline [225]. Large observational studies have demonstrated that urinary DKK3 levels predict vulnerability to AKI after cardiac surgery, serving as a biomarker capable of anticipating AKI before its occurrence [226]. Furthermore, baseline uDKK3/uSCr potentially identify patients with high risk of AKI and persistent renal impairment under invasive coronary and peripheral procedures [227–229].

#### Inflammatory biomarkers

Inflammation is a key driver of renal maladaptation, with some cytokines and chemokines serving as critical indicators for assessing acute injury and chronic functional deterioration. Among these, interleukin-18 (IL-18) serves as a critical bridge between innate immunity and structural injury. Primarily produced and synthesized in monocytes, macrophages, and PTCs, precursor IL-18 is cleaved by caspase-1 into its bioactive mature form during renal injury. This activation exacerbates ischemic damage by recruiting neutrophils and other immune effectors [230, 231]. Mechanistically, genetic deletion of IL-18 reduces tubular necrosis and impede the AKI-CKD transition by inhibiting macrophage polarization [232]. Clinically, urinary IL-18 (uIL-18) is a highly sensitive early biomarker, typically rising prior to SCr changes [233]. Its diagnostic utility is further enhanced when used in combination with other markers. In patients with cirrhosis, uIL-18 combined with NGAL effectively differentiates acute tubular necrosis from other renal dysfunctions and predicts mortality [234]. Similar predictive value is observed in the cardiac setting, where the dynamic fluctuations of uIL-18 and gelsolin (GSN) correlate with persistent injury following catheterization [235]. However, while uIL-18 shows higher predictive efficacy in pediatric cohorts, the significant variability in reported cut-off values, ranging from 6.39 to 125 pg/ml, underscores the need for standardized thresholds in clinical practice [236]. Monocyte chemoattractant protein-1 (MCP-1) mediates macrophage infiltration, and its elevation in plasma and urine is a well-established hallmark of AKI [237]. In postoperative cardiac surgery cohorts, the ratio of urinary epidermal growth factor (EGF) to MCP-1 has emerged as a critical prognostic indicator; while higher MCP-1 levels correlate with an increased risk of CKD, elevated EGF, reflecting tubular regenerative capacity, serves as a protective marker against chronic progression [238]. Secreted by monocytes and macrophages, CCL14 recruits immune cells and act as a direct profibrotic stimulus [239]. Large observational studies confirm that serum CCL14 levels accurately identify patients with moderate-to-severe AKI who are at risk for non-recovery and those who may ultimately require renal replacement therapy [240–244]. Together, these inflammatory mediators provide a multidimensional view of the renal microenvironment, offering critical insights into the likelihood of successful repair versus chronic progression.

### Emerging biomarkers for predicting AKI-CKD transition

Increased proteinuria indicates persistent injury and failed repair in AKI-CKD transition. Although post-AKI proteinuria is common among hospitalized patients and often transient, its new onset or persistent worsening carries distinct CKD risk characteristics closely associated with long-term adverse renal outcomes [245]. The urinary albumin-to-creatinine ratio (ACR) assessed three months after AKI, combined with eGFR, more effectively predicts subsequent rapid decline in renal function than the AKI event itself or its severity, demonstrating superior discriminatory ability in risk stratification [246]. Indeed, late-stage elevation of plasma KIM-1 and urinary NGAL (uNGAL) also indicates their utility as markers for persistent renal injury following AKI [215, 247]. In a cohort study with a median follow-up of 4.3 years, persistent elevation of urinary KIM-1, MCP-1, and plasma tumor necrosis factor receptor 1 (TNFR1) was associated with a 2 to threefold increased risk of CKD onset or progression, whereas elevated urinary Uromodulin (UMOD) was linked to a significantly reduced risk [248]. uNGAL is associated with persistent AKI, and incorporating uNGAL into predictive models moderately improves prognostic prediction [249].

A prospective cohort study covering Canada and the United States, involving 865 cardiac surgery patients, demonstrated that elevated postoperative urinary EGF levels significantly indicate a low risk of CKD onset or progression, whereas elevated urinary MCP-1 levels independently predicted long-term adverse CKD outcomes [238]. Urinary insulin-like growth factor-binding protein-7 (IGFBP-1) levels were not significantly elevated in patients progressing to KDIGO Stage 2 AKI but increased markedly in those progressing to Stage 3 AKI, requiring renal replacement therapy, or dying within 30 days postoperatively. This suggests IGFBP-1 can specifically identify high-risk populations for severe AKI with poor clinical outcomes, outperforming markers that solely reflect injury [250]. Serum circulating TNFR1/2 levels significantly increase after renal injury, and their combination with suPAR aids in assessing risk in COVID-19 patients with complicated AKI [251]. Serum circulationg TNFR1/2 levels reflect the severity of renal injury, and their sustained elevation indicates ongoing tissue damage and renal fibrosis in AKI-CKD transition [252]. In CKD patients, in-hospital AKI onset correlates with increased plasma TNFR1, TNFR2, and KIM-1 levels post-hospitalization, with AKI potentially accelerating renal function decline months to years after acute injury [253]. A multivariate model incorporating soluble tumour necrosis factor receptor 1 (sTNFR1), sTNFR2, cystatin C, and eGFR, measured 3 months post-hospitalization in AKI patients, was developed to estimate the risk of new-onset or worsening CKD after AKI, achieving an area under the curve (AUC) of 0.79 [254].

Angiotensinogen (AGT) is a key indicator for assessing the activation status of the renal renin-angiotensin system (RAS) system in AKI [255]. Studies indicate that elevated urinary AGT (uAGT) levels indicate poor AKI outcomes in ICU patients [256, 257]. In follow-up studies of patients with acute tubular necrosis, those who progressed from AKI to CKD exhibited significantly higher uAGT levels at 3 months post-biopsy compared to those who recovered. This demonstrates uAGT utility in dynamically monitoring renal structural recovery and treatment response [258]. Moreover, serum AGT has excellent predictive value in patients with acute myocardial infarction (AMI). Studies show serum AGT achieves AUCs of 0.811 and 0.700 for predicting AKI and CKD progression, respectively, suggesting its potential to identify high-risk patients. Integrating it into clinical decision algorithms could optimize early intervention and management strategies [256]. Cytokeratin 20 (CK20) is a novel biomarker significantly upregulated in damaged proximal TECs. Urinary CK20 (uCK20) exhibits superior predictive accuracy for the AKI-CKD transition and effectively refines risk stratification across diverse clinical etiologies, including cardiac surgery and heart failure, revealing its potential clinical utility in AKI of multiple causes [259]. Urinary decoy receptor 2 (uDcR2), a cleaved type II transmembrane protein, independently predicts poor renal recovery following AKI with superior diagnostic accuracy compared to [TIMP-2] ·[IGFBP-7], particularly when integrated with clinical and pathological variables to refine prognostic risk stratification [260]. FGF23 is an osteogenic hormone that maintains serum phosphorus homeostasis by promoting renal phosphorus excretion and suppressing 1,25(OH)₂D production. Under pathological conditions, renal glycerol-3-phosphate (G-3-P) upregulates FGF23 levels via the glycerol-3-phosphate acyltransferase (GPAT)/lysophosphatidic acid (LPA)/lysophosphatidic acid receptor 1 (LPAR1) pathway, which is especially pivotal in acute kidney damage [261]. A recent study indicates serum FGF23 appears more promising for assessing AKI severity [262]. Early postoperative measurements of C-terminal fibroblast growth factor 23 (cFGF23) and intact fibroblast growth factor 23 (iFGF23) correlate with AKI following cardiac surgery [263]. However, FGF23 variations are mostly driven by mineral metabolism and inflammatory dysregulation instead of acute or chronic characteristics of renal injury, making its predictive value controversial [264]. Table 1 provides a summary of potential biomarkers in AKI-CKD transition with their clinical application and limitations.

**Table 1:** An overview of potential biomarkers in AKI-CKD transition

Source	Biomarker	Clinical application	Limitations	References
Serum	TNFR1/2	Persistent elevation indicates ongoing kidney damage and renal fibrosis	Lacks explanation of the molecular mechanism for release in kidney injury	[252]
Serum	CD35	Identifies subclinical AKI; predicts persistent AKI, mortality, and progression to AKD	Specific for podocyte injury in sepsis-associated AKI, not for ischemic AKI	[265]
Urine	ACR	Higher levels after AKI are related to higher risk of CKD progression	Single measurement at 3 months post-discharge; ignores dynamic patterns or long-term prognosis	[246]
Urine	CK20	Early prediction of severe AKI and progression to CKD outperforms traditional biomarkers	Requires dynamic monitoring; insufficient validation of specificity; unclear pathophysiological mechanism	[259, 266, 267]
Urine	DcR2	Independently predicts risk of kidney non-recovery and fibrosis	Small sample size; lacks validation in prospective studies	[260]
Urine	EGF/Cr	Higher levels urinary EGF/Cr are connected with decreased CKD risk and reduced MAKE risk	Insufficient sample representativeness; CKD definition based on single measurements; lacks follow-up data	[238, 268, 269]
Urine	MCP-1	Higher levels after cardiac surgery are associated with a higher risk of CKD	Subjects predominantly white males; lacks data on biomarker evolution in follow-up cohort	[238]
Urine	DKK3/Cr	Predicts AKI occurrence and identifies high-risk patients prone to CKD or kidney failure	Limited to European populations; association with renal replacement therapy cannot be inferred	[226]
Urine	NGAL	Enables very early diagnosis; predicts dialysis needs, mortality, and AKI-CKD transition	Specificity reduced due to influence from systemic inflammation	[249]
Urine/Serum	AGT	Assesses structural recovery; predicts AKI-CKD progression and response to RAS therapy	Limited to preclinical animal models; lacks large-scale clinical data support	[258]

*AKI* acute kidney injury, *AKD* acute kidney disease, *CKD* chronic kidney disease, *TNFR1/2* soluble tumor necrosis factor receptor 1/2, *CD35* complement receptor 1, *ACR* albumin-to-creatinine ratio, *CK20* cytokeratin 20, *DcR2* decoy receptor 2, *EGF* epidermal growth factor, *EGF/Cr* epidermal growth factor to creatinine ratio, *MCP-1* monocyte chemoattractant protein 1, *DKK3* urinary Dickkopf-related protein 3, *NGAL* neutrophil gelatinase-associated lipocalin, *AGT* angiotensinogen, *RAS* renin-angiotensin system, *MAKE* major adverse kidney events

### Imaging and omics technologies

In clinical practice, traditional imaging modalities such as ultrasound, computed tomography (CT), and magnetic resonance imaging (MRI) are employed for AKI diagnosis and risk assessment based on hemodynamic abnormalities [270]. While conventional imaging techniques can visualize renal anatomy, they face limitations in early AKI diagnosis due to low spatiotemporal resolution and radiation exposure. Moreover, they fail to provide molecular level information, making it difficult to reveal early pathological changes in AKI. Fibrosis is an adverse effect of AKI and the most accurate indicator of CKD development. Biopsy remains the sole approach for precisely evaluating the severity of renal fibrosis. Emerging functional and molecular imaging technologies have enabled a transition from macroscopic structural assessment to microscopic molecular and physiological levels [271, 272]. Molecular and functional imaging allow non-invasive and internal observation of particular biological activities. This not only enhances diagnostic accuracy but also provides critical technical support for ultra-early warning and dynamic monitoring of renal fibrosis progression [273].

Molecular imaging combines non-invasive imaging techniques with molecular probes or contrast agents to detect and quantify specific physiological or pathophysiological processes. In the early phase of injury, stimulus-responsive nanoplatforms utilize early injury markers like microRNA-21 to trigger dual-mode fluorescence and photoacoustic imaging, enabling earlier detection of sepsis-associated AKI than current diagnostic standards [274]. P-selectin binding peptide (PBP)-engineered EVs (PBP-EVs) exhibit enhanced selectivity toward injured kidneys, combining imaging and therapeutic functions with potential for early, real-time monitoring of renal injury severity [275]. In the progression of chronic kidney disease, extracellular matrix proteins present a promising non-invasive and specific imaging technique for evaluating renal fibrosis. The elastin-specific magnetic resonance imaging agent (ESMA) facilitates non-invasive and repeatable assessment of renal fibrosis, enabling both longitudinal monitoring of anti-fibrotic effectiveness and detection of fibrosis progression after early reversible injury, prior to the alteration of conventional functional indicators [276]. Fibroblast activation protein (FAP) -specific PET/CT imaging identifies active fibrotic processes to track maladaptive repair. It significantly outperforms conventional ultrasound or MRI in predicting fibrosis 14 days post-AKI [277]. Research demonstrates that cation-ferritin-enhanced MRI (CFE-MRI) can accurately and non-invasively identify pathological heterogeneity in AKI-CKD transition by quantitatively assessing microscopic changes in glomerular volume, density, and spatial distribution [278].

Functional MRI (fMRI) monitors renal tissue characteristics due to its significant advantages: non-invasiveness, absence of ionizing radiation, lack of depth limitations, and high soft-tissue resolution. Currently, multiple fMRI techniques are widely applied in the assessment of AKI. Arterial spin labeling (ASL) and intravoxel incoherent motion (IVIM) enable in-depth analysis of the renal vasculature and perfusion function [279]. Blood oxygen level-dependent (BOLD) is used to evaluate tissue oxygenation status [280]. Meanwhile, diffusion tensor imaging (DTI), diffusion kurtosis imaging (DKI), T1/T2 mapping, and quantitative susceptibility-weighted imaging (QSM) reflect structural alterations and fibrosis formation [281, 282]. Given the lack of curative interventions to reverse CKD progression, clinical survival heavily relies on early prognostic assessment during the AKI phase and precise interventions during recovery. Studies indicate that dynamic changes in renal volume (TKV) in AKI patients correlate closely with chronic subclinical damage [283]. One year after AKI, although SCr levels returned to baseline in all participants, abnormal TKV reduction in some patients foreshadowed potential CKD progression [283, 284]. Elevated cortical T1 mapping values and loss of corticomedullary differentiation (CMD) positively correlate with eGFR decline and renal volume loss [285]. High T1 mapping indicates increased inflammation and/or edema, serving as a key indicator of poor repair assessment [286]. Conversely, low T2 values based on BOLD sequences reflect greater hypoxia [287], while parenchymal T2 mapping primarily correlates with tubular epithelial edema [288]. Furthermore, early cortical perfusion impairment measured by ASL significantly correlates with subsequent renal atrophy [284], further substantiating the predictive role of hemodynamic impairment in AKI outcomes.

High-throughput omics technologies, including genomics, transcriptomics, proteomics, epigenomics, metabolomics, and lipidomics, are propelling kidney research into the molecular realm [265, 289]. Urine proteomics/peptidomes aid in identifying specific biomarkers, enabling non-invasive detection of early CKD and renal interstitial fibrosis. The CKD273 classifier, consisting of 273 urinary peptides, has shown potential for early prediction value of CKD in non-albuminuria individuals [290]. Findings from the SomaScan platform indicate that plasma proteins such as osteopontin and tenascin C are significantly correlated with tubular injury and assess AKI-CKD transition [291]. In a study paralleling cardiac surgery patients with marathon runner cohorts, researchers found that dynamic changes in the plasma proteome closely correlated with the transcriptome profile of non-adaptive repair in the proximal TECs and effectively predicted postoperative AKI progression and subsequent renal atrophy [292]. Furthermore, CD35 levels in urinary EVs were demonstrated to identify subclinical AKI and predict AKD progression [265]. Another metabolome-wide association study of 5,087 German CKD patients identified 55 urinary metabolites significantly correlated with adverse renal outcomes and/or mortality [293]. However, due to high testing costs, complex sample preparation procedures, and data heterogeneity across cohorts, the clinical translation of these biomarkers is significant challenged, including the standardization of assays, the complexity of biological interpretation, and insufficient validation of pathological relevance.

The ideal diagnostic approach for tracking AKI-CKD progression in the future should combine functional and molecular imaging with multi-omics indicators. The former offers location, qualitative, and semi-quantitative data, facilitating in vivo viewing of pathophysiological processes. The latter elucidates fundamental mechanisms from a systems biology viewpoint and provides easily measurable liquid-based markers for dynamic evaluation.

## Potential therapeutic interventions to attenuate the AKI-CKD transition

**Fig. 6:**
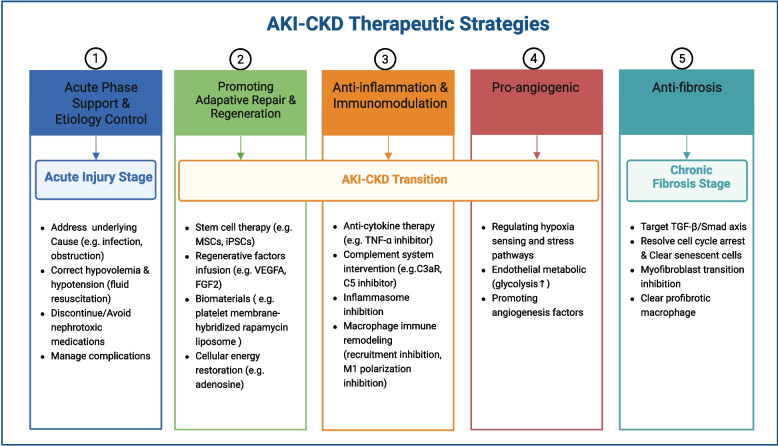
Potential therapeutic strategies in AKI-CKD transition. Contemporary treatments for AKI and CKD primarily focus on etiological management and hemodynamic stabilization during the acute phase to avert the aggravation of initial damage. Consequently, the therapy for AKI-CKD transition emphasizes the promotion of tissue repair and immunomodulation, aiming to inhibit chronic fibrotic processes by regulating inflammation and improving of microcirculatory perfusion. Focusing on physiological anti-fibrotic pathways and the targeted elimination of profibrotic cells has become an intriguing strategy to mitigate the development of renal interstitial fibrosis. MSC mesenchymal stem cell; iPSCs induced pluripotent stem cells; VEGFA vascular endothelial growth factor A; FGF2 fibroblast growth factor 2; TNF-α tumor necrosis factor alpha; C5 complement component 5; C3aR complement component 3a receptor. Figure created with BioRender.com

The AKI-CKD transition is influenced by multiple risk factors, including injury characteristics, baseline renal function, and systemic comorbidities [4, 6, 294]. Current clinical strategies of AKI are primarily supportive, encompassing etiological interventions, to prevent further injury, and management of AKI complications [2]. Measures to preserve renal function involve rectifying hypovolemia and hypotension to ensure hemodynamic stability, as well as rigorously reducing or avoiding nephrotoxic agents [295, 296]. In conditions of primary glomerular or interstitial disorders, corticosteroid medication can be utilized alongside other treatments as considered suitable. Concurrently, blood pressure and glucose control, alongside RAS inhibitors or SGLT2i, reduce proteinuria and delay functional decline [297]. Among approved medications, SGLT2i demonstrates broad application potential. Recent studies indicate that SGLT2i can mitigate early injury by improving renal hemodynamics and restoring mitochondrial function, exerting protective effects during the AKI-CKD progression through sustained anti-fibrotic actions [298]. They also effectively reduce urinary albumin and uromodulin excretion [299]. Starting renal replacement therapy at the right time is critical for patients with progressive organ failure or persistent AKI [296, 300, 301]. Nevertheless, clinical interventions remain limited for early, precise intervention in AKI and preventing its chronic progression. Here, we summarize current clinical trials on the treatment in AKI-CKD transition in Table 2. As research into AKI-CKD deepens, novel intervention targets are emerging. Anti-inflammatory and antioxidant agents, inhibitors of TECs G2/M arrest, pro-angiogenic factors, epigenetic therapies, tissue regeneration promoters, and fibrosis inhibitors are being explored (Fig. 6).

**Table 2:** An overview of recent and ongoing clinical trials of treatment in AKI-CKD transition

Agents	Disease Type	Sample size	Target	Phase	NCT Number	Completion Status
AST-120	AKD, CKD	100	Uremic Toxin Absorbent	4	NCT07182422	Recruiting
QRX-3	CKD	3000	NADPH Oxidase Modulation	2, 3	NCT06866236	Not yet recruiting
GDC-8264	AKI, CKD	404	RIPK1 inhibitor	2	NCT06602453	Recruiting
Dapagliflozin; Empagliflozin; Canagliflozin	AKI, CKD	264	SGLT2i	2	NCT06528405	Recruiting
Dapagliflozin	AKI, CKD	100	SGLT2i	2, 3	NCT05713851	Recruiting
Iron dextran	AKI, CKD	120	Iron dextran	2	NCT05960227	Completed
Ravulizumab	AKI, CKD	736	C5 inhibitor	3	NCT05746559	Active not Recruiting
Azacitidine	AKI, CKD	60	DNA methyltransferase inhibitor	1, 2	NCT05325099	Recruiting
Irbesartan	AKI, CKD	508	ARB	3	NCT05272878	Active not Recruiting
Vitamin B Complex	AKI, CKD	260	Vitamin B Complex	4	NCT04893733	Completed
20-25% Albumin fluid	AKI, CKD	856	Albumin	4	NCT04705896	Recruiting
Low protein diet + Ketosteril	AKI, CKD	50	N/A	N/A	NCT02831062	Completed

Data extracted from clinicaltrials.gov

*AKI* acute kidney injury, *AKD* acute kidney disease, *CKD* chronic kidney disease, *SGLT2i* sodium-glucose cotransporter 2 inhibitors, *NADPH* nicotinamide adenine dinucleotide phosphate hydrogen, *RIPK1* receptor-interacting serine/threonine-protein kinase 1, *C5* complement component 5, *ARB* angiotensin II receptor blocker, *DNA* deoxyribonucleic acid, *NCT* National Clinical Trial identifier, *N/A* not applicable

### Promoting adaptive repair and regeneration

The reparative capacity of the kidney following AKI largely determines the ultimate outcome of the disease. The primary aim of the recovery procedure is to restore the structural and functional integrity of renal tissue. Currently, stem cell therapies, particularly the application of mesenchymal stem cells (MSCs), demonstrate significant therapeutic potential due to immunomodulation, anti-inflammation and importantly promoting regeneration [302]. MSCs promote repair by paracrine release of growth factors including EGF, hepatocyte growth factor (HGF), that stimulate tubular cell proliferation. They also release miR-125b-5p via exosomes, which targets p53 protein expression in TECs to induce cell cycle arrest and apoptosis, thereby counteracting fibrosis, inflammation, and hypoxia [303]. However, despite the promising prospects, the clinical translation of MSC therapies still faces major challenges including low retention rates, limited survival in injured tissue, and insufficient production of therapeutic factors within the renal environment. Innovative approaches including genetic engineering and pretreatment with drugs and cell factors to improve MSC efficacy are being actively explored with the goal to overcome these restrictions [304]. For example, pretreating stem cells with Wnt pathway activators further enhances their reparative effects [305]. Astilbin pretreatment enhances MSC efficacy in AKI and AKI-CKD mice by inducing polarization of macrophages towards an M2-like phenotype via prostaglandin-endoperoxide synthase 2 (PTGS2)-mediated pathways [306]. Another study developed a novel medium named CFY, enabling large-scale expansion of human induced pluripotent stem cell-derived nephrogenic progenitor cells (hiPSC-NPCs). These cells and their secreted VEGF-A were demonstrated to effectively alleviate AKI and CKD in mice, offering a new cellular therapy in renal diseases [307].

Beyond stem cells, other strategies have also shown potential to promote repair. In preclinical studies, early exogenous supplementation of fibroblast growth factor 2 (FGF2) following AKI onset significantly alleviated post-injury interstitial fibrosis [308]. Furthermore, a Phase I/II clinical trial pioneered the use of autologous peripheral blood CD34 + cells mobilized by granulocyte colony-stimulating factor and immobilized for the treatment of severe AKI, employing a dose-escalation protocol. The study found that systemic transplantation of a proregenerative subset derived from peripheral blood mononuclear cells (PBMCs) promoted renal function recovery by suppressing tissue fibrosis and delivering anti-inflammatory, proangiogenic, and anti-apoptotic microRNA (miRNAs) to ischemic tissues [309]. A platelet membrane (PM)-hybridized rapamycin liposome, where rapamycin showed promising potential as an anti-inflammatory therapeutic agent in AKI interventions. While platelet membrane proteins synergistically achieve long-term tissue repair by promoting the recruitment and differentiation of hematopoietic stem cells and endothelial progenitor cells within CD34 + cells, enhancing their adhesion to damaged tissues, and upregulating stromal cell-derived factor-1 (SDF-1) levels in IRI kidney [310]. However, constrained by systemic delivery efficiency, integrating biomaterials to achieve precise and efficient targeted interventions is considered to have greater application potential. Besides, research indicates that severe AKI can induce mitochondrial DNA mutations in renal cells, impairing respiratory chain function and leading to severe ATP synthesis deficiency. This energy deprivation renders cells incapable of repair and stress response. Supplementing with adenosine, an ATP precursor, effectively restores cellular energy metabolism and function, offering a novel energy supplementation strategy to promote renal injury repair [311].

### Anti-inflammatory and immunomodulatory strategies

Persistent chronic inflammation driven by the innate immune system is considered one of the critical mechanisms driving AKI-CKD transition. Current therapeutic strategies primarily focus on inhibiting proinflammatory cytokines, blocking abnormal complement system activation, and reshaping macrophage phenotypes through immunomodulation to create a microenvironment conducive to tissue repair. Etanercept, a TNF-α inhibitor, can alleviate renal inflammation and fibrosis by reducing soluble TNF released from profibrotic immune cells [312], and reduces TNF-α levels and partially improves renal injury in an adenine-induced CKD animal model [313]. Compared to EGFR inhibitors like erlotinib, which suppress TNF production by inhibiting cell recruitment, direct targeting of TNF-α may offer advantages regarding potential adverse effects [312]. Nonetheless, the systemic implications of these therapies on renal function must be taken into account. In the proximal TECs, IL-1βR1 is activated and inhibits apolipoprotein M (ApoM), consequently worsening AKI. Conversely, they provide renal protection by sustaining VEGF-A expression and maintaining vascular endothelial cell density [314, 315]. Besides, modulating the CCL2-CCR2 axis with CCR2 inhibitors or PC3-secreted microprotein (PSMP)-neutralizing antibodies, or blocking S100A8/A9-mediated intercellular interactions, to diminish the recruitment and accumulation of pro-inflammatory monocyte-derived macrophages constitutes a pivotal therapeutic approach for alleviating post-AKI inflammation and preventing its advancement to fibrosis [316]. EVs from adipose-derived MSCs can alleviate M1 polarization of macrophagein renal and mitigate cisplatin-induced AKI via the TXNIP-IKKα/NF-κB signaling pathway [317]. Although the complement component 3a receptor (C3aR) inhibitor SB290157 has shown potential for renal inflammation and fibrosis, its dual C3aR/C5aR2 agonist activity has hindered its clinical translation [318]. Clinical study is investigating whether prophylactic terminal complement inhibition with C5 inhibitor ravulizumab reduces AKI or other adverse renal events in CKD patients undergoing cardiac surgery [319]. Li et al. have comprehensively detailed targeted therapies for pyroptosis in AKI, summarizing pyrophosphate inhibitors targeting the NLRP3 inflammasome, cystatin, and Gasdermin D (GSDMD) [320, 321]. However, due to the complex spatiotemporal and dose heterogeneity of NLRP3 inflammasome activation in AKI-CKD, therapeutic strategies must shift from blind direct inhibition to precision modulation based on disease stage, cellular microenvironment, and individual characteristics. Beyond interventions targeting inflammatory mediators and the complement pathway, strategies targeting phenotypic remodeling of inflammatory cell are gaining attention.

### Anti-fibrotic strategies

Irreversible renal fibrosis represents the ultimate outcome of progressive AKI. When tubular repair fails, activated fibroblasts are transformed into myofibroblasts, inducing massive ECM deposition. Current anti-fibrotic therapeutic strategies predominantly center on the TGF-β/Smad signaling cascade, the central regulatory axis governing renal fibrogenesis. Additionally, interventions targeting EMT, EndMT and MMT, alongside alleviating tubular cell cycle arrest and cellular senescence, serves as indispensable complementary interventions for fibrosis attenuation.

TGF-β1 plays a central driving role in renal fibrosis, and therapeutic strategies targeting this pathway have been extensively explored. Current research directions primarily include inhibiting TGF-β expression or biological activity, developing TGF-β receptor and Smad3 inhibitors, using anti-TGF-β neutralizing antibodies, and targeting in upstream regulatory factors or interacting proteins. Multiple small molecules and biologic drugs exert anti-fibrotic effects by directly or indirectly modulating the TGF-β pathway. The P2Y12 inhibitor Clopidogrel may inhibit MMT conversion via the TGF-β/Smad3 pathway, emerging as an efficient anti-fibrotic medication for CKD [322]. Polyphenol-nicotinamide adenine dinucleotide (NAD) + nanoparticles arrest AKI-CKD progression by restoring Sirt1-mediated homeostasis and suppressing TGF-β-driven fibrosis [323]. Targeting key nodes in the TGF-β signaling network represents another effective strategy. IRI induces nuclear Smad3/β-catenin complex formation, while melatonin and poricoic acid A (PAA) interfere with Smad3-β-catenin interaction. Supplementing PAA enhances melatonin’s inhibitory effect on TGF-β/Smad and Wnt/β-catenin pathways [324]. Annexin A13 (ANXA13) directly binds the intracellular domain of transforming growth factor-β receptor 1 (TGF-βR1) and prevents its phosphorylation; this process leads to the inactivation of Smad3 signaling pathway, thereby inhibiting renal TECs apoptosis [325]. The aforementioned multi-level research findings collectively confirm that blocking the TGF-β signaling pathway is a crucial strategy for managing CKD progression.

Severe injury causes TECs to arrest in the G2/M phase, resulting in sustained release of profibrotic cytokines and fibrosis. Therefore, resolving cell cycle arrest is key to breaking this vicious cycle. Research indicates that normal cyclical proliferation can be restored by p53 inhibitors [25]. As a high-affinity selective class IIa histone deacetylase (HDAC) inhibitor, TMP195 significantly alleviates tubular atrophy, interstitial fibrosis, and inflammatory responses in aristolochic acid nephropathy (AAN) mice by relieving G2/M phase arrest in TECs [326]. Cisplatin-induced subcellular translocation of mouse double minute 2 homolog (MDM2) in tubules triggers G2/M arrest by upregulating p53, synergistically promoting integrin β8 degradation and TGF-β1 activation [327]. Human serum albumin-thioredoxin fusion protein (HSA-Trx) significantly blocked AKI-CKD transition by inhibiting G2/M arrest and apoptosis in tubular cells while effectively modulating oxidative stress and inflammation, demonstrating excellent renal protective potential [328].

Irreversible cell cycle arrest leads to cell senescence, which further expedite AKI-CKD progression by altering the microenvironment through their secreted factors. The removal of senescent cells or the inhibition of senescence has become a highly promising therapy. Intermittent D + Q treatment helps to mitigate renal aging and slow down the advancement of renal fibrosis. Thus, senolytic therapy could serve as a promising strategy for treating fibrosis [329]. While CDK4/6 inhibitor Palbociclib reverses the anti-fibrotic effects of Dasatinib combined with Quercetin in eliminating senescent cells [330]. Targeting CD38 released by macrophages with abnormal NAD + metabolism significantly suppresses the induced renal tubular senescence and fibrosis [331]. Acute administration of the MCL-1-specific inhibitor UMI-77 effectively reduces tubular senescence and fibrosis compared to ABT-263 treatment [332]. Procyanidin C1 (PCC1) alleviates renal interstitial fibrosis by eliminating senescent TECs and thus attenuating fibroblast activation by reducing SASP levels [333]. Furthermore, dexmedetomidine (Dex) effectively inhibits renal TECs senescence and inflammation by activating adrenergic receptors, thereby mitigating AKI-CKD transition [334].

Targeting specific cell subsets within the profibrotic renal microenvironment, including profibrotic TECs subpopulations and immune cell subsets, particularly profibrotic macrophages, can remodel the renal microenvironment and attenuate fibrogenic signaling. Chen et.al. developed a bio-activated self-assembling peptide (BIVA-PK) that induces FN1 + SPP1 + Mrc1 + macrophage death and functional alteration, remodeling the renal immune microenvironment to block AKI-CKD transition, highlighting the therapeutic promise of targeting macrophages in CKD [335]. Besides, adoptive cellular immunotherapy offers a novel approach for directly eliminating fibrotic effector cells. Furthermore, chimeric antigen receptor T cell (CAR-T) cell therapy targeting PDGFRβ has demonstrated superior efficacy compared to FAP CAR-T in renal fibrosis. By directly targeting ECM-producing cells, this approach provides a strategy for clinical translation in AKI-CKD [336].

### Pro-angiogenic and metabolic strategies

Post-AKI capillary rarefaction leads to prolonged chronic hypoxia in the kidney, contributing to CKD [337]. Hypoxia stabilizes HIF-1α/2α, upregulates glycolysis, inhibits mitochondrial respiration, and increases fatty acid synthase expression [338]. Potential AKI-CKD intervention strategies include targeting HIF and Nuclear factor erythroid 2-related factor 2 (Nrf2), or regulating VEGF and its receptors, or improving endothelial cell metabolism to maintain microvascular density. Research indicates that focusing on the hypoxia response and metabolic reprogramming in endothelial cells is a vital therapeutic strategy. The post-ischemic silencing of prolyl hydroxylase domain protein 1/2/3 (PHD1/2/3) in endothelial cells impairs renal recovery by impacting genes associated with hypoxia and glycolysis. Inhibition of glycolysis using the monocarboxylate transporter 4 (MCT4) inhibitor syringopine leads to adaptive repair in PHD-deficient mice, indicating renal endothelial glycolysis as an attractive therapeutic target [339]. Isolated PHD2 deficiency also suppresses proinflammatory gene expression and inflammatory cell recruitment via HIF-1-dependent mechanisms [340]. Ginsenoside Rb1 promotes angiogenesis by activating the VEGFR2/AKT pathway, mitigating AKI-induced capillary rarefaction and providing a possible strategy to impede CKD progression [341]. A recent study demonstrated that FG4592 treatment significantly alleviated renal fibrosis and improved renal vascular regeneration. This effect may result from the activation of the HIF-1α/VEGF-A/VEGFR1 signaling pathways and the promotion of the endogenous antioxidant superoxide dismutase 2 (SOD2) expression [342]. It is noteworthy that VEGF-A intervention exhibits time-phase dependence. Early supplementation with VEGF-A prevents renal injury by protecting microvascular structure and counteracting secondary tubular hypoxic damage, while late-stage anti-VEGF-A therapy attenuates renal fibrosis progression [343].

## Conclusion and future perspectives

The AKI-CKD transition represents a critical global health challenge [4]. Recent breakthroughs in single-cell and multi-omics technologies have fundamentally reshaped our understanding of the AKI-CKD transition, revealing intricate cellular heterogeneity, dynamic cell–cell interactions, and microenvironmental remodeling [344]. We comprehensively delineate the core pathology of the AKI-CKD transition, involving sustained G2/M arrest, cellular senescence, formation of an inflammatory microenvironment, fibroblast activation, and microvascular rarefaction [345–347]. These cells contribute to the fibrotic response by activating resident fibroblasts and immune cells through the persistent induction of the TGF-β/Smad, Wnt/β-catenin, and Hedgehog signaling, which collectively drive myofibroblast differentiation and excessive extracellular matrix deposition. Furthermore, chronic inflammation mediated by the NLRP3 inflammasome and NF-κB pathways, alongside microvascular rarefaction and resulting hypoxia, establishes a self-perpetuating cycle of tissue injury that ultimately leads to end-stage renal fibrosis. Recent breakthroughs have increasingly highlighted the roles of metabolic remodeling and epigenetic memory in reinforcing maladaptive, profibrotic phenotypes. Current research perspectives have shifted from the dysfunction of isolated cell populations toward a more integrated understanding of functional niches. These include maladaptive repair niches associated with chronic injury, profibrotic niches influenced by fibroblast subtypes, and tissue-destructive niches [105, 348]. By integrating artificial intelligence, single-cell multi-omics, and spatial metabolomics, researchers aim to construct high-resolution atlases of renal repair and fibrosis [349]. However, findings derived from these advanced technologies require cautious interpretation and rigorous experimental validation to effectively connect fundamental discoveries with clinical application.

In the realm of diagnosis, the field is undergoing a paradigm shift from delayed functional markers toward precise molecular phenotyping. Although SCr and urine output remain the cornerstones of clinical diagnosis, their limitations in predicting long-term outcomes necessitate the use of non-invasive biomarkers such as KIM-1, NGAL, and [TIMP-2] ·[IGFBP-7] [350]. Concurrently, molecular and functional imaging techniques have surpassed the anatomical constraints of macroscopic structures to achieve real-time, non-invasive assessment of renal oxygenation, perfusion, tissue edema, and the window of fibrotic activity. By integrating high-throughput multi-omics technologies such as the CKD273 urinary peptide classifier, SomaScan proteomics, and metabolome-wide association studies, researchers can comprehensively reveal the heterogeneity of AKI outcomes from genetic variation to metabolic products. However, clinical application still faces challenges in standardization, threshold unification, and the depth of association with pathological mechanisms. Future efforts should focus on constructing multimodal, integrated, precision early-warning systems that leverage artificial intelligence and machine learning techniques to intricately combine multi-omics and radiomics data, thereby establishing dynamic and individualized risk-prediction models [350].

Clinical management of AKI and CKD remains primarily focused on etiological intervention and supportive care. Although RAS blockers and SGLT2i offer cardiorenal benefits, they cannot reverse established fibrosis. Furthermore, KDIGO guidelines do not recommend RAS inhibitors for patients with AKI, and timely cessation within two days of AKI onset in regular users is often suggested to reduce short-term mortality risk [351, 352]. Consequently, we highlight innovative interventions targeting adaptive repair, anti-inflammation, and antifibrosis. However, certain strategies may also exert context-dependent effects depending on the stage of the AKI-CKD transition [353]. For example, the FOXO4-DRI inhibitory peptide effectively clears p16 high senescent cells during early stages without immediate functional improvement, whereas delayed clearance effectively attenuates fibrosis and promotes repair. Such findings necessitate precise timing for therapeutic intervention [354]. Future therapeutic paradigms should shift from single-target blockade toward temporally precise interventions and multi-target synergistic regulation. Based on dynamic molecular imaging or biomarker monitoring, interventions should be administered within specific therapeutic windows such as early-stage anti-inflammation, mid-stage repair promotion, and late-stage targeting of specific fibrotic pathways. Research should explore combination therapies that pair senolytic drugs to clear senescent cells with anti-fibrotic or immunomodulatory strategies. Nanomedicine-based strategies offer a novel pathway for high-efficiency and precise intervention in AKI to prevent CKD progression through nanoparticle-mediated intrinsic therapy, targeted drug delivery, inhibition of pathogenic processes, and intelligent modulation of pharmacokinetics [355]. Concurrently, well-designed prospective clinical trials are required to thoroughly assess the long-lasting effectiveness and safety of traditional herbal formulas, natural products, novel biologics, and innovative cell therapies.

Existing rodent models effectively simulate acute, short-term AKI but remain limited in their ability to capture the long-term chronic consequences or the heterogeneous progression observed in complex clinical contexts such as diabetes and aging [356]. Furthermore, clinical renal biopsies are rarely performed during the initial stages of acute injury, which complicates the correlation between preclinical data and human pathology [357]. The advancement of innovative technologies such as organoids will be instrumental in deepening understanding of human-specific cell injury and repair mechanisms [9]. There is a pressing necessity to validate preclinical biomarkers and therapeutic targets using animal models that better reflect the range of human diseases. This includes aged models, metabolic comorbidity models, and human-derived organoids, to enhance the clinical translatability from rodents to humans. AKI should be viewed not merely as a localized renal event but as a systemic trigger that induces dysfunction in distant organs such as the heart, lungs, and liver through inflammatory, metabolic, and hemodynamic disturbances [109, 358, 359]. Future research aims to adopt a systemic organ network perspective and utilize network analysis to evaluate multi-organ homeostatic imbalance. This approach will facilitate the exploration of synergistic intervention strategies for conditions such as cardiorenal syndrome, ultimately achieving holistic and systemic therapeutic outcomes.

In summary, AKI-CKD transition is a self-perpetuating cycle driven by persistent injury signals and a degrading microenvironment. As we move towards the age of precision nephrology, the integration of cutting-edge technologies, from AI-assisted diagnosis to biomaterial scaffolds and stem cell therapy, holds the promise of halting this vicious cycle. Future efforts must focus on standardizing novel biomarkers and validating stage-specific interventions, ultimately aiming to achieve precise interruption of the AKI-CKD continuum.

## Data Availability

Not applicable.

## Abbreviations

AAN: Aristolochic acid nephropathy
ACR: Albumin-to-creatinine ratio
AKD: Acute kidney disease
AKI: Acute kidney injury
AKT: Protein kinase B
AMI: Acute myocardial infarction
AMPK: AMP-activated protein kinase
ANXA13: Annexin A13
AP-1: Activator protein 1
APC: Adenomatous polyposis coli protein
ApoM: Apolipoprotein M
AREG: Amphiregulin
Arg1: Arginase 1
ASC: Apoptosis-associated speck-like protein containing a CARD
ASL: Arterial spin labelling
ATAC-seq: Assay for transposase-accessible chromatin using sequencing
ATF3: Activating transcription factor 3
ATP: Adenosine triphosphate
AUC: Area under the curve
Axin: Axin protein
BIVA-PK: Bio-activated self-assembling peptide
BNIP3: BCL2 interacting protein 3
BOLD: Blood oxygen level-dependent imaging
BH4: Tetrahydrobiopterin
CS: Citrate synthase
C3: Complement component 3
cGAS: Cyclic GMP-AMP synthase
C3aR: Complement component 3a receptor
C5a: Complement component 5a
C5aR2: Complement component 5a receptor 2
CAR-T: Chimeric antigen receptor T cell
CCL2/5/6/8/9: C-C motif chemokine ligand 2/5/6/8/9
CG1: Cyclin G1
CCR1/2/5: C-C motif chemokine receptor 1/2/5
CD300a: CD300a molecule
CDK: Cyclin-dependent kinase
CDK1/4/5/6: Cyclin-dependent kinase 1/4/5/6
CFE-MRI: Cation-ferritin-enhanced magnetic resonance imaging
circRNA: Circular RNA
c-JUN: Jun proto-oncogene
CK1α: Casein kinase 1α
CK20: Cytokeratin 20
CKD: Chronic kidney disease
CKD273: Chronic kidney disease 273 urinary peptide classifier
CLCF1: Cardiotrophin-like cytokine factor 1
CMD: Corticomedullary differentiation
CPT1/2: Carnitine palmitoyltransferase 1/2
CRLF1: Cytokine receptor-like factor 1
CT: Computed tomography
CTGF: Connective tissue growth factor
Cx43: Connexin 43
CXCL2,3,4,16: C-X-C motif chemokine ligand 2,3,4,16
CXCL-iFibro: CXCL-expressing inflammatory fibroblast
CXCR6: C-X-C motif chemokine receptor 6
DAMP: Damage-associated molecular pattern
DC: Dendritic cell
DcR2: Decoy receptor 2
DHH: Desert hedgehog
DKI: Diffusion kurtosis imaging
DTI: Diffusion tensor imaging
ECM: Extracellular matrix
EGF: Epidermal growth factor
EGFR: Epidermal growth factor receptor
EMT: Epithelial–mesenchymal transition
EndMT: Endothelial–mesenchymal transition
eNOS: Endothelial nitric oxide synthase
ERBB4-IR: Erbb4 intronic region
ERK: Extracellular signal-regulated kinase
ERK1/2: Extracellular signal-regulated kinase 1/2
ESMA: Elastin-specific magnetic resonance agent
ESRD: End-stage renal disease
ESRRA: Estrogen-related receptor α
EV: Extracellular vesicle
EZH2: Enhancer of zeste homolog 2
FA: Fatty acid
FAO: Fatty acid β-oxidation
FAP: Fibroblast activation protein
Fas: Fas cell surface death receptor
FasL: Fas ligand
FDA: Food and Drug Administration
FGF2: Fibroblast growth factor 2
FGF23: Fibroblast growth factor 23
FIP1: Factor interacting with poly(A) polymerase 1
fMRI: Functional magnetic resonance imaging
FN1: Fibronectin 1
FOLR2: Folate receptor β
FOXO4-DRI: Fork head box O transcription factor 4-D-Retro-Inverso
FR-PTC: Failed-repair proximal tubular cell
G1: Gap 1 cell cycle phase
G2/M: G2/M cell cycle phase
G-3-P: Glycerol-3-phosphate
GADD45: Growth arrest and DNA damage-inducible 45
GFR: Glomerular filtration rate
Gli1: Glioma-associated oncogene homolog
GM-CSF: Granulocyte–macrophage colony-stimulating factor
GPAT: Glycerol-3-phosphate acyltransferase
GPX4: Glutathione peroxidase 4
GSDMD: Gasdermin D
GSDME: Gasdermin E
GSK-3β: Glycogen synthase kinase-3β
GSN: Gelsolin
GTPCH-I: GTP cyclohydrolase 1
HDAC: Histone deacetylase
HGF: Hepatocyte growth factor
HIF-1α: Hypoxia-inducible factor 1α
HIF-2α: Hypoxia-inducible factor 2α
hiPSC-NPC: Human induced pluripotent stem cell-derived nephrogenic progenitor cell
HK: Hexokinase
HK2: Hexokinase 2
HMGB1: High-mobility group box 1
HOXA5: Homeobox A5
Hoxc8: Homeobox C8
HSA-Trx: Human serum albumin–thioredoxin fusion protein
ICU: Intensive care unit
cFGF23: C-terminal fibroblast growth factor 23
iFGF23: Intact fibroblast growth factor 23
IFNAR1: Interferon-α/β receptor subunit 1
IFN-β: Interferon-β
IFN-γ: Interferon-γ
IGFBP-1: Insulin-like growth factor-binding protein 1
IGFBP-7: Insulin-like growth factor-binding protein 7
IHH: Indian hedgehog
IKKα: Inhibitor of nuclear factor-κB kinase subunit α
IL-1β: Interleukin-1β
IL-1βR1: Interleukin-1β receptor 1
IL-4/5/10/13/17/18/22/33: Interleukin-4/5/10/13/17/18/22/33
ILC1/2/3: Type 1/2/3 innate lymphoid cell
iPT: Inflammation-associated proximal tubule cell
IRI: Ischaemia–reperfusion injury
ISG15: Interferon-stimulated gene 15
IVIM: Intravoxel incoherent motion
Jag1: Jagged 1
JNK: C-Jun N-terminal kinase
KIM-1: Kidney injury molecule 1
KLF6: Krüppel-like factor 6
KRM: Kidney resident macrophage
LDH: Lactate dehydrogenase
LEF: Lymphoid enhancer-binding factor
L-FABP: Liver-type fatty acid-binding protein
lncRNA: Long non-coding RNA
lnc-TSI: Long non-coding RNA-TGF-β signalling inhibitor
LPA: Lysophosphatidic acid
LPAR1: Lysophosphatidic acid receptor 1
LRIG1: Leucine-rich repeats and immunoglobulin-like domains protein 1
LRP: Low-density lipoprotein receptor-related protein
m6A: N6-methyladenosine
Magi1: Membrane-associated guanylate kinase, WW and PDZ domain-containing protein 1
MALAT1: Metastasis-associated lung adenocarcinoma transcript 1
MAPK: Mitogen-activated protein kinase
MCL-1: Myeloid cell leukaemia 1
MCP-1: Monocyte chemoattractant protein 1
MCT4: Monocarboxylate transporter 4
MDM2: Mouse double minute 2 homolog
METTL3: Methyltransferase-like 3
Mincle: Macrophage inducible C-type lectin
miRNA: MicroRNA
Mmp12: Matrix metalloproteinase 12
MMT: Macrophage-myofibroblast transition
Mrc1: Mannose receptor C-type 1
MRI: Magnetic resonance imaging
mROS: Mitochondrial reactive oxygen species
MSCs: Mesenchymal stem cells
mtDNA: Mitochondrial DNA
NAD: Nicotinamide adenine dinucleotide
NEK7: NIMA-related kinase 7
NET: Neutrophil extracellular trap
NFAT5: Nuclear factor of activated T cells 5
NF-κB: Nuclear factor-κB
NGAL: Neutrophil gelatinase-associated lipocalin
NID1: Nidogen 1
NKT cell: Natural killer T cell
NLRP3: NOD-like receptor family pyrin domain-containing 3
Notch3: Notch receptor 3
Nrf2: Nuclear factor erythroid 2-related factor 2
ns-MRA: Non-steroidal mineralocorticoid receptor antagonist
Orai1: ORAI calcium release-activated calcium channel protein 1
p16: Cyclin-dependent kinase inhibitor 2A
p27: Cyclin-dependent kinase inhibitor 1B
p21: Cyclin-dependent kinase inhibitor 1A
PAA: poricoic acid A
PCC1: Procyanidin C1
P2X7: P2X purinoceptor 7
P2Y12: Purinergic receptor P2Y12
PAI-1: Plasminogen activator inhibitor 1
PAMP: Pathogen-associated molecular pattern
PANX1: Pannexin 1
PBMC: Peripheral blood mononuclear cell
PBP: P-selectin-binding peptide
PBP-EVs: P-selectin-binding peptide-engineered EVs
PCNA: Proliferating cell nuclear antigen
PDGF: Platelet-derived growth factor
PDGFRβ: Platelet-derived growth factor receptor-β
PDIA3: Protein disulfide isomerase family A member 3
pEMT: Partial epithelial-mesenchymal transition
PET/CT: Positron emission tomography-computed tomography
Pfkfb3: 6-Phosphofructokinase/fructose-2,6-bisphosphatase 3
PHD: Prolyl hydroxylase domain protein
PI3K: Phosphoinositide 3-kinase
PKM2: Pyruvate kinase M2
PM: Platelet membrane
PPAR-α: Peroxisome proliferator-activated receptor-α
PROM1: Prominin 1
PSMP: PC3-secreted microprotein
PTCH: Patched receptor
P-TEFb: Positive transcription elongation factor b
PTEN: Phosphatase and tensin homolog
PTGS2: Prostaglandin-endoperoxide synthase 2
PUM2: Pumilio RNA binding family member 2
QSM: Quantitative susceptibility mapping
RAAS: Renin angiotensin aldosterone system
Rapgef5: Rap guanine nucleotide exchange factor 5
RAS: Renin-angiotensin system
RB1: Retinoblastoma protein
ROS: Reactive oxygen species
RUNX1: Runt-related transcription factor 1
RXRα: Retinoid X receptor α
SARA: Smad anchor for receptor activation
SASP: Senescence-associated secretory phenotype
SCr: Serum creatinine
SDC1: Syndecan 1
SDC4: Syndecan 4
SDF-1: Stromal cell-derived factor 1
Sema3: Semaphorin 3
SGK3: Serum/glucocorticoid-regulated kinase 3
SGLT2i: Sodium–glucose cotransporter 2 inhibitor
SHH: Sonic hedgehog
Siglec-F: Sialic acid-binding immunoglobulin-like lectin F
SIRT1: Sirtuin 1
Smad: Suppressor of mothers against decapentaplegic homologue
Smad2/3/4/7: Suppressor of mothers against decapentaplegic homolog 2/3/4/7
SMO: Smoothened receptor
Smurf2: Smad-specific E3 ubiquitin protein ligase 2
SNAI1: Snail family transcriptional repressor 1
SOD2: Superoxide dismutase 2
SOX9: SRY-box transcription factor 9
SPP1: Secreted phosphoprotein 1
ST2: Suppression of Tumorigenicity 2
STAT3: Signal transducer and activator of transcription 3
STC: Scattered tubular cell
STING: Stimulator of interferon genes
sTNFR1/2: Soluble tumour necrosis factor receptor 1/2
SUFU: Suppressor of fused homolog
suPAR: Soluble urokinase-type plasminogen activator receptor
TASC: Autophagy–space coupling structure
TCA cycle: Tricarboxylic acid cycle
TCF: T cell factor
NADPH: Nicotinamide adenine dinucleotide phosphate hydrogen
TEC: Tubular epithelial cell
TGF-β1/2: Transforming growth factor-β1/2
TGFβ-R1/2: TGF-β receptor type 1/2
Th1/2/17: T helper 1 cell 1/2/17
THBS1: Thrombospondin 1
Tie2: Tyrosine kinase receptor
TIMP-2: Tissue inhibitor of metalloproteinases 2
TKV: Total kidney volume
TLRs: Toll-like receptors
TLR2/4: Toll-like receptor 2/4
TMAO: Trimethylamine N-oxide
TNC: Tenascin C
TNF: Tumour necrosis factor
TNFR1/2: Tumour necrosis factor receptor 1/2
TNF-α: Tumour necrosis factor-α
TOPK: T-LAK cell-originated protein kinase
Treg: Regulatory T cell
TXNIP: Thioredoxin-interacting protein
AGT: Angiotensinogen
uCK20: Urinary cytokeratin 20
Cr: Creatinine
uDKK3: Urinary Dickkopf-related protein 3
uEGF: Urinary epidermal growth factor
uIL-18: Urinary interleukin-18
UMOD: Uromodulin
uNGAL: Urinary neutrophil gelatinase-associated lipocalin
UTR: Untranslated region
UUO: Unilateral ureteral obstruction
VCAM1: Vascular cell adhesion molecule 1
VEGF: Vascular endothelial growth factor
VEGF-A: Vascular endothelial growth factor A
VEGFR1: Vascular endothelial growth factor receptor 1
VEGFR2: Vascular endothelial growth factor receptor 2
VIM: Vimentin
VNN1: Vanin 1
Wnt: Wingless-type MMTV integration site family
WNT5A: Wnt family member 5A
WT1: Wilms’ tumour 1
YAP/YAP1: Yes-associated protein/Yes-associated protein 1
YME1L: YME1-like mitochondrial metalloprotease
α-SMA: α-smooth muscle actin
